# Supplementary material for: Interplay between ATRX and IDH1 mutations governs innate immune responses in diffuse gliomas
Source: Nat Commun. 2024 Jan 25;15:730. doi: 10.1038/s41467-024-44932-w (PMC10810843; doi:10.1038/s41467-024-44932-w)
Supplement: Supplementary file 1 — Supplementary Information [file 41467_2024_44932_MOESM1_ESM.pdf]

Supplementary information

Interplay between *ATRX* and *IDH1* mutations governs innate immune responses in diffuse gliomas

Hariharan, S., Whitfield, B. T., *et al*.

Supplementary figure 1

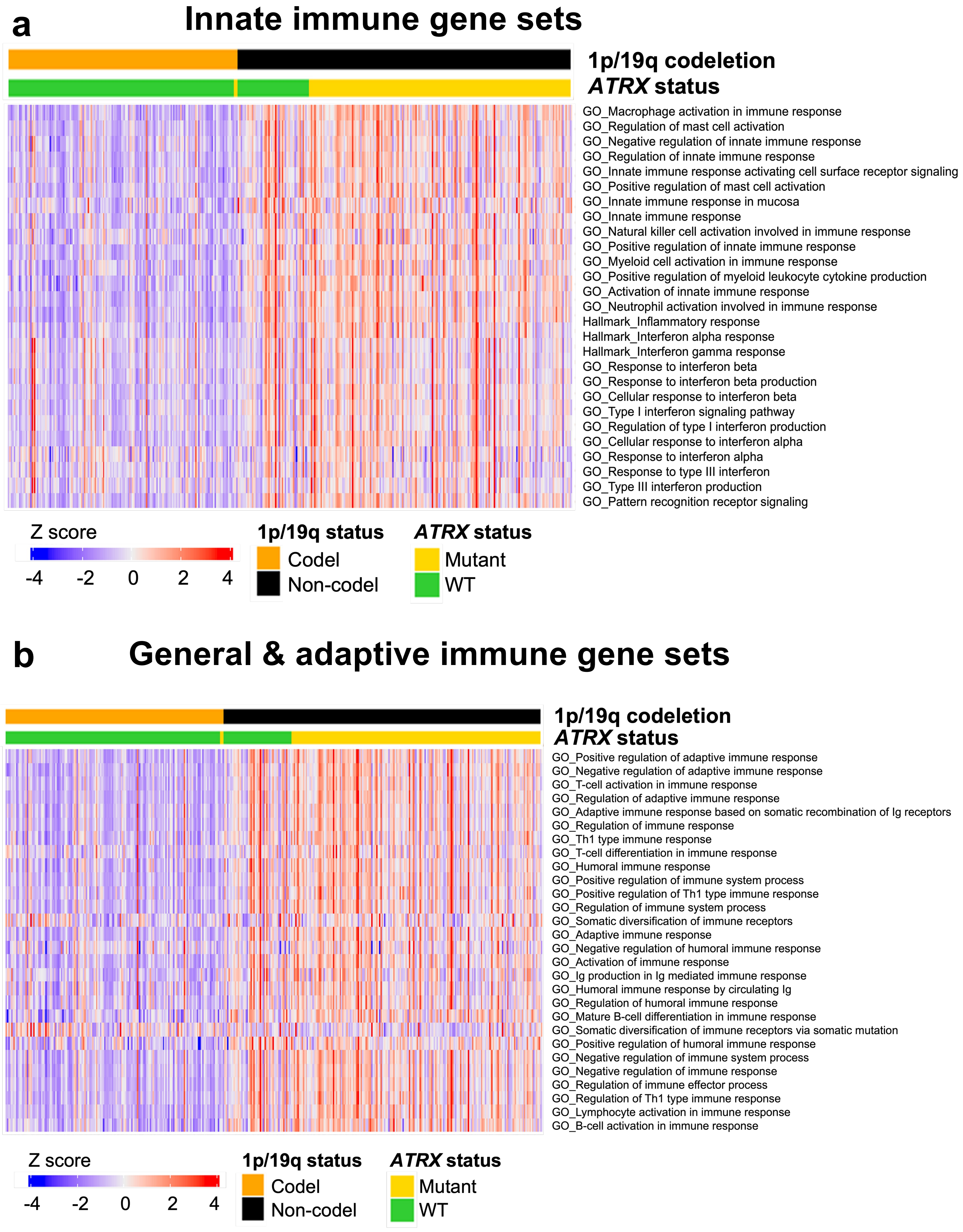

Supplementary figure 1 – supporting figure 1a. Astrocytomas are immunologically engaged compared to oligodendrogliomas.

(**a, b**) Heatmap from ssGSEA showing enrichment for various innate immune-related (**a**) and general and adaptive immune-related (**b**) gene sets from CNS/Brain TCGA LGG PanCancer Atlas Study, comparing 1p-19q noncodel/ *IDH* mutant/ *ATRX*-mutant astrocytomas (n=191) to 1p-19q codel/ *IDH* mutant/ *ATRX*-WT oligodendrogliomas (n=164). A relatively smaller number of 1p-19q codel/ *IDH* mutant/ *ATRX*-mutant (n=3) and 1p-19q noncodel/ *IDH* mutant/ *ATRX* WT (n=52) are also included in the analysis for comparison.

Supplementary figure 2

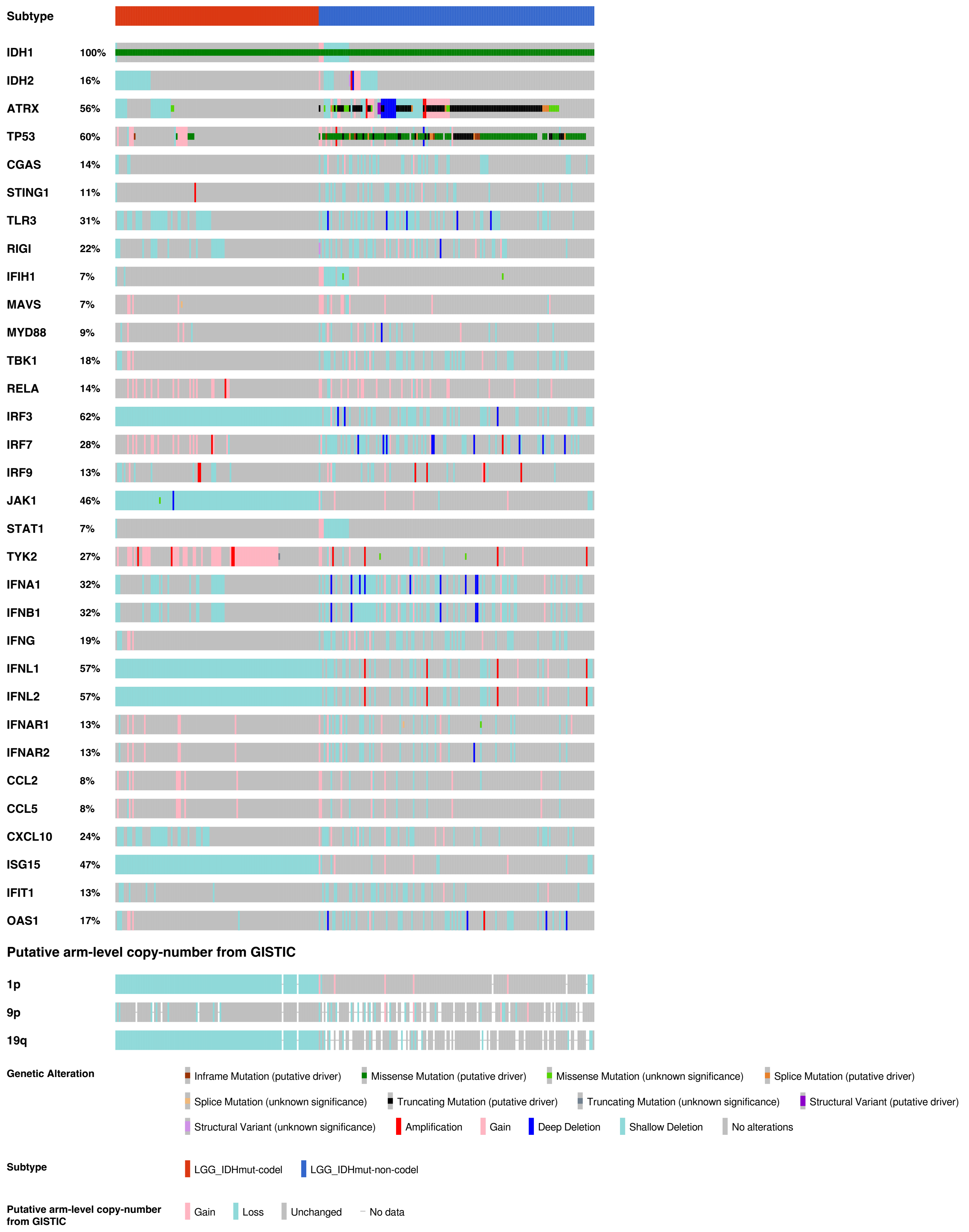

Supplementary figure 2 – supporting figures 1a and b. *IDH*-mut astrocytomas exhibit lower frequency of genetic alterations in immune-related genes compared to *IDH*-mut oligodendrogliomas. Oncoprint output from cBioportal for the CNS/Brain TCGA LGG PanCancer Atlas Study analyzing *IDH* mutant-LGGs for mutation status of indicated genes. Datasets from *IDH*-mutant 1p/19q codel subtype are compared with *IDH*-mutant 1p/19q noncodel subtype.

Supplementary figure 3

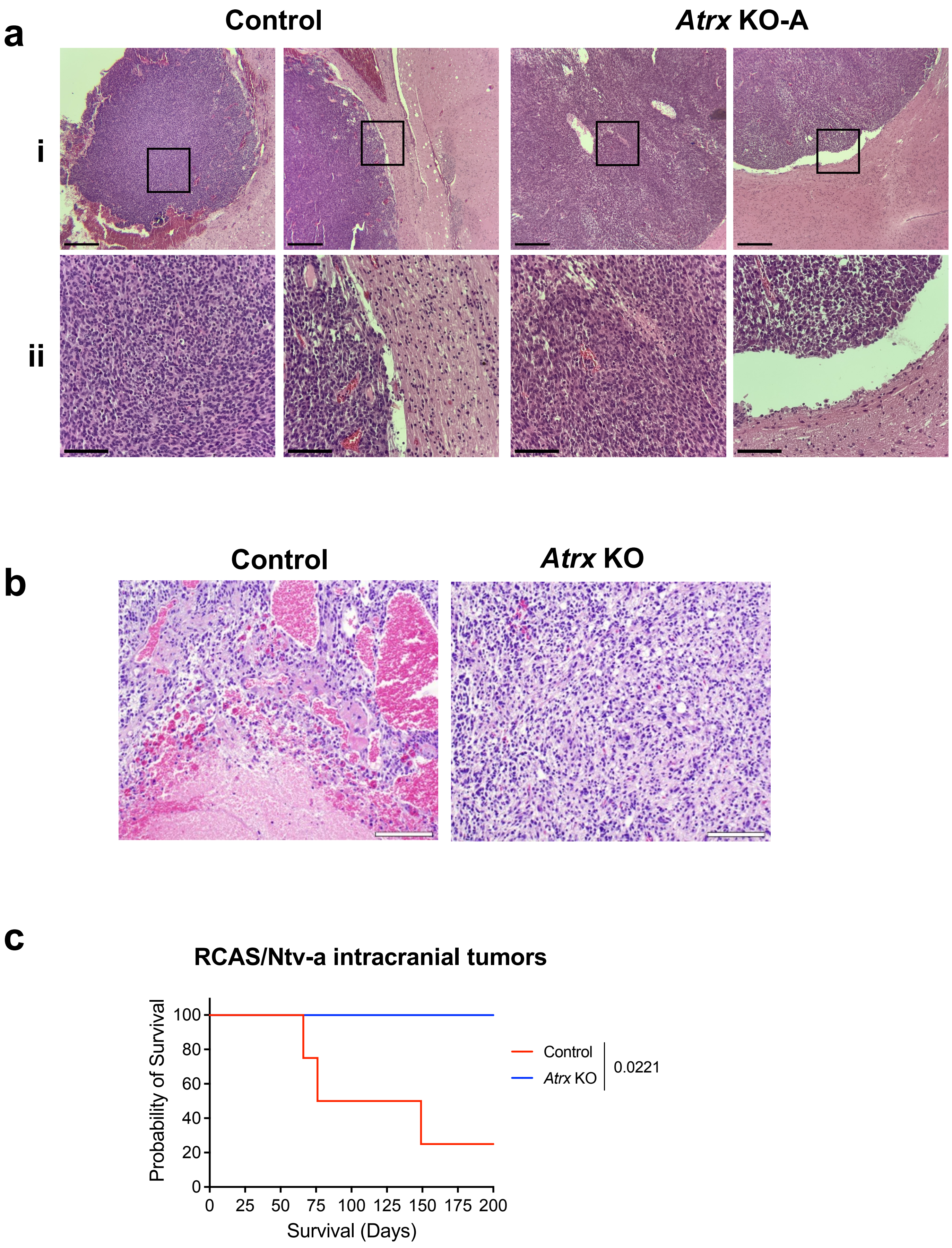

Supplementary figure 3

d

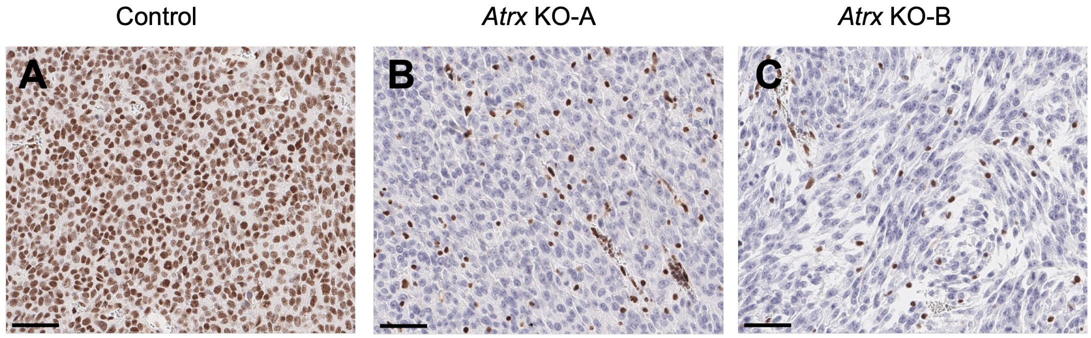

e

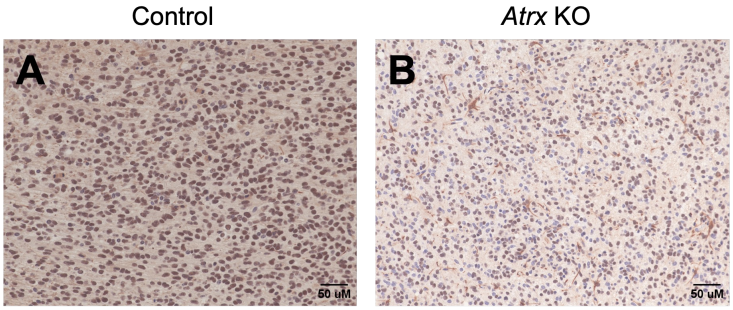

Supplementary figure 3 – supporting figures 2a and b. Histology from *Atrx*^{WT} and *Atrx*-KO CT2A and RCAS/Ntv-a tumors.

(**a**) H&E staining performed on end-stage CT2A CRISPR control (*Atrx*^{WT}) and *Atrx* KO-A tumors. Row **i** represents images taken using a 4x objective (Scale bar: 400µm); Row **ii** represents images taken using a 20X objective of the insets shown in row **i**. (Scale bar: 100µm). Number of brains subjected to IHC per group: n=3 for CRISPR control and *Atrx* KO-A. (**b**) H&E staining performed on end-stage *Atrx*$^{+/+}$ (Control) or *Atrx*$^{-/-}$ (*Atrx* KO) tumors. Scale bar: 100µm. Number of brains subjected to IHC per group: n=3 for Control and *Atrx* KO. **(c)** Kaplan Meir survival curves for C57BL/6 mice bearing intracranial RCAS/ Ntv-a *Atrx*$^{fl/fl}$ (*Atrx* KO) (n=5) and *Atrx*$^{+/+}$ (Ctrl) (n=4) tumors generated by re-injecting *Atrx*$^{+/+}$ (Ctrl) or *Atrx*$^{fl/fl}$ (*Atrx* KO) cell lines derived from the de-novo model. P-value of 0.0221 represents group comparison calculated using log-rank test. (d) Representative IHC images for ATRX expression (brown) in CT2A CRISPR Control (*Atrx*^{WT}) (A), *Atrx* KO-A (B) and KO-B (C) end-stage tumors, with hematoxylin counter-staining (blue). Scale bar: 50µm. Number of brains subjected to IHC per group: n=3 for CRISPR control, *Atrx* KO-A and *Atrx* KO-B. (e) Representative IHC images for ATRX expression in RCAS/Ntv-a *Atrx*^{WT} (A) and *Atrx* KO (B) end-stage tumors. Scale bar: 50µm. Number of brains subjected to IHC per group: n=3 for Control and *Atrx* KO. Source data are provided as a Source Data file.

Supplementary figure 4

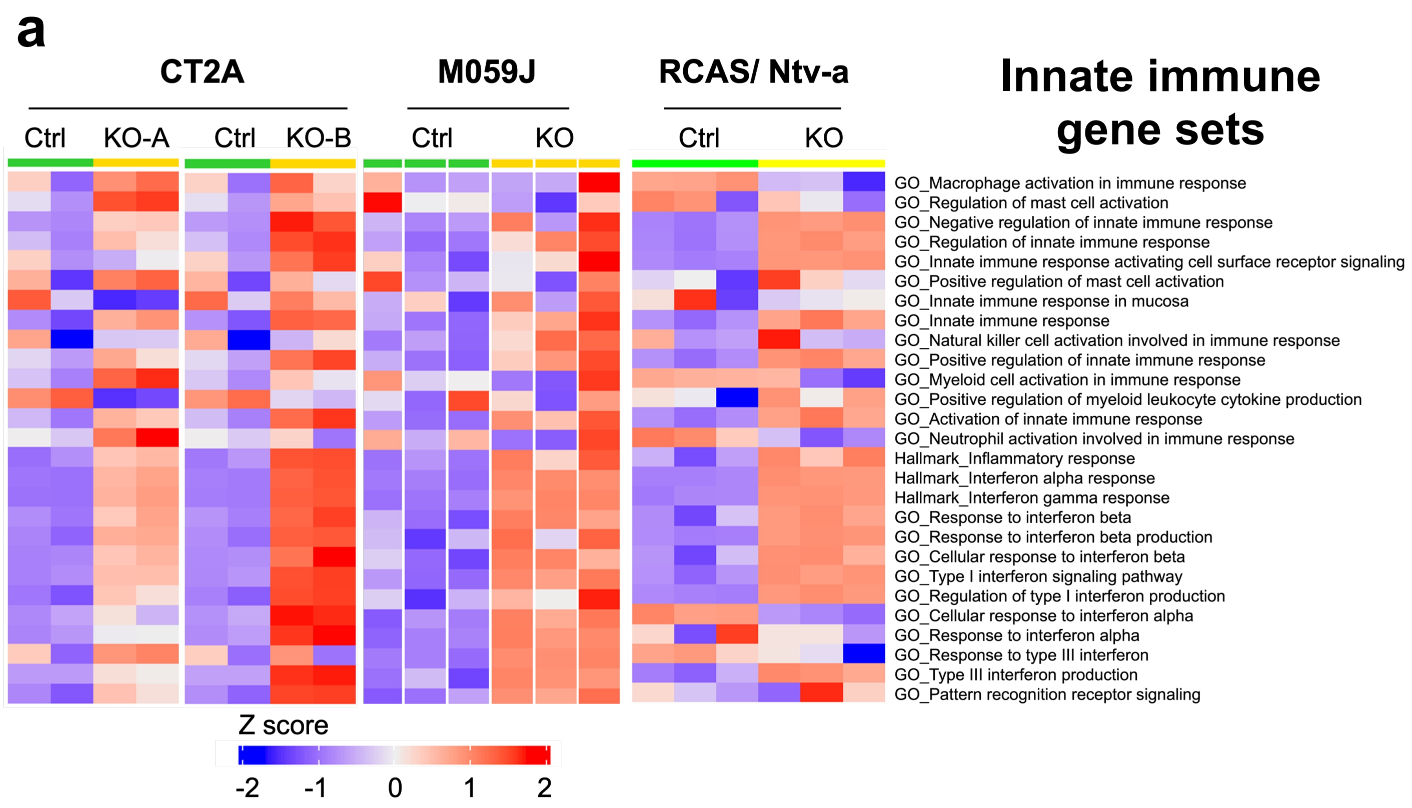

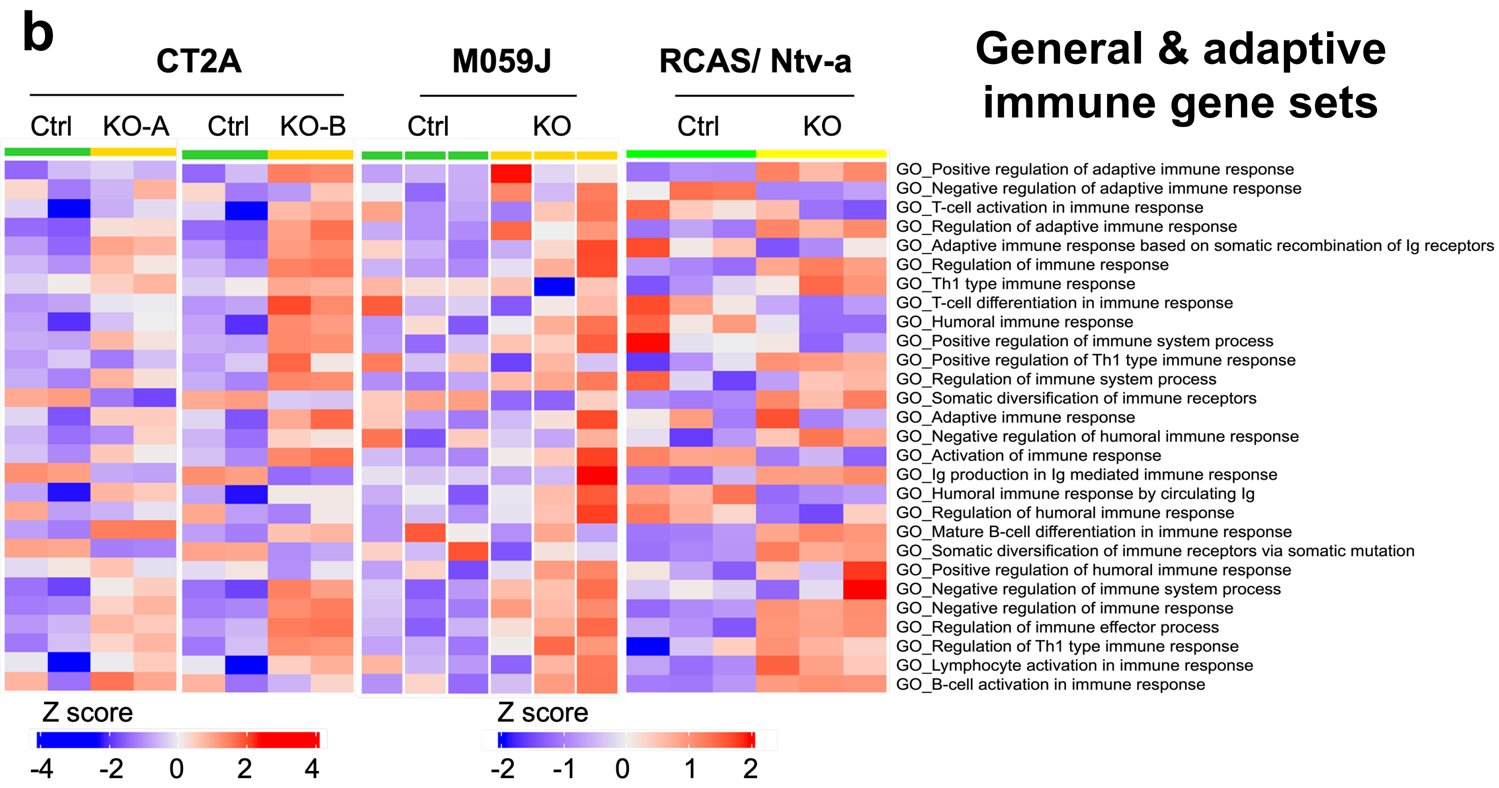

Supplementary figure 4 – supporting figure 2d. ATRX loss is associated with a pro-inflammatory signaling.
(**a, b**) Heatmap from ssGSEA showing enrichment for various innate immune-related (**a**) and general and adaptive immune-related (**b**) gene sets in CT2A *Atrx* KO-A and KO-B clones, M059J *ATRX* KO cells and RCAS/Ntv-a *Atrx* KO compared to their respective *ATRX*^{WT} counterparts. n=2 technical replicates per cell line for CT2A expression data; n= 3 technical replicates per cell line for M059J expression data; n=3 biological replicates (3 consecutive cell passages) per cell line for RCAS/Ntv-a cell line expression data.

Supplementary figure 5

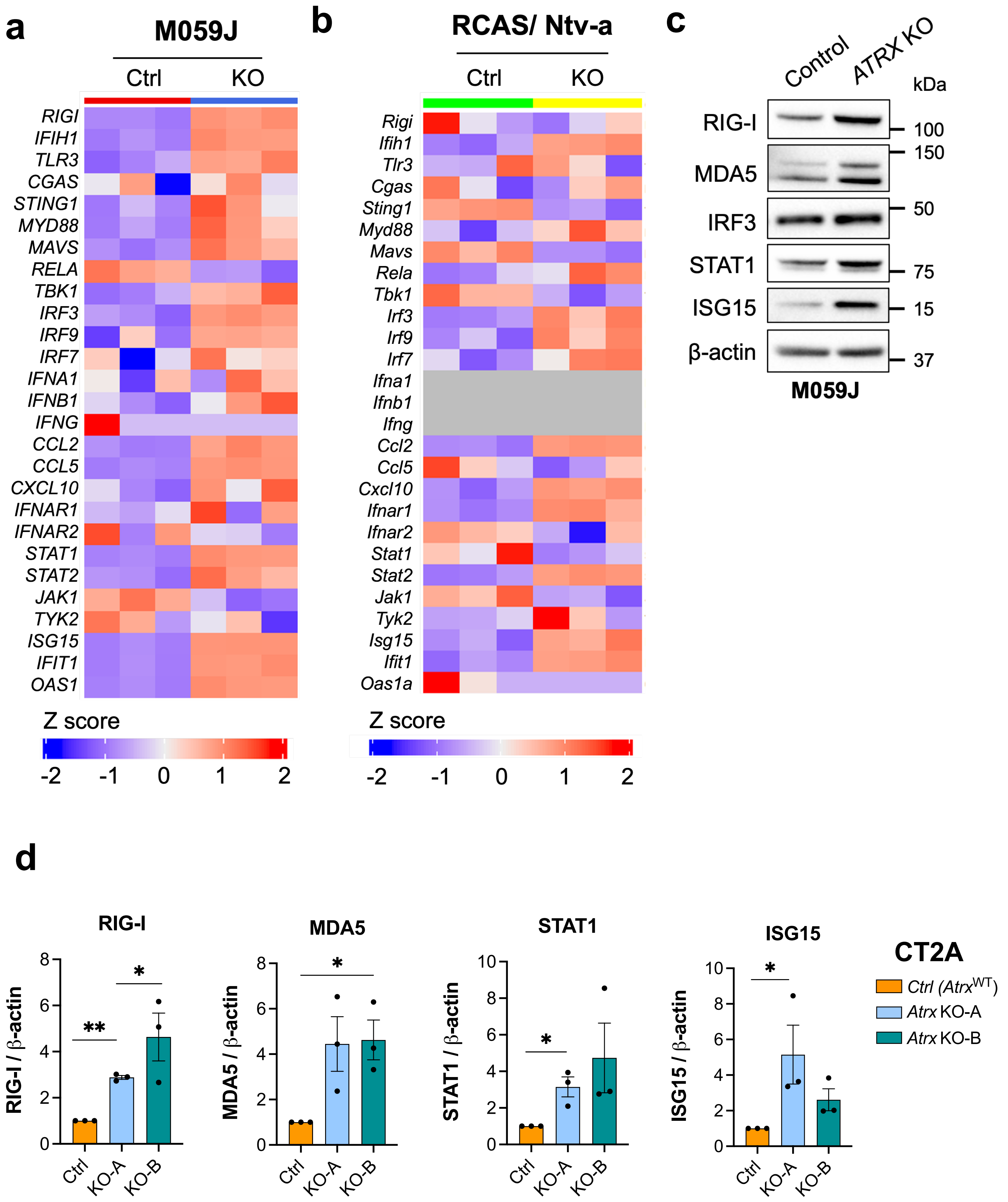

Supplementary figure 5

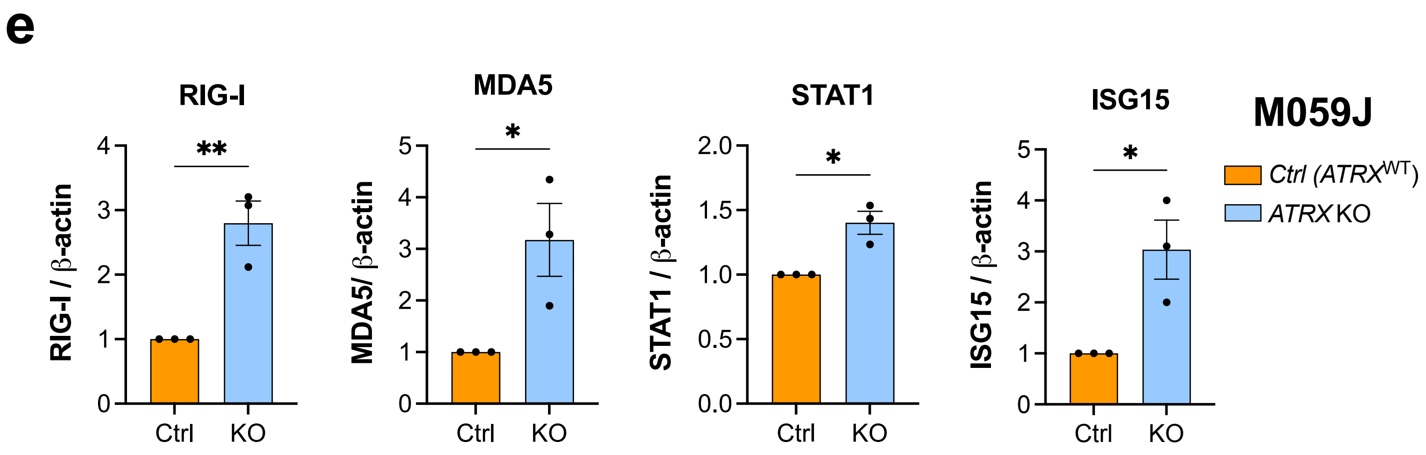

Supplementary figure 5 – supporting figures 3a and b. ATRX loss is associated with increased baseline gene and protein expression.
(**a, b)** Heatmap showing differential expression of immune-related genes in M059J CRISPR control (Ctrl) and *ATRX* KO cells (**a**) and RCAS/Ntv-a *Atrx*$^{+/+}$ (Ctrl) and *Atrx*$^{-/-}$ (KO) cells (**b**). n= 3 technical replicates per cell line for M059J expression data; n=3 consecutive cell passages per cell line for RCAS/Ntv-a cell line expression data. (**c**) Representative western blot using lysates from M059J CRISPR ctrl (Ctrl) and *ATRX* KO cells (KO) screened for proteins involved in innate immune signaling. β-actin serves as the loading control. N=3 independent experiments. Densitometry values are indicated in Supplementary Fig. 5e.(**d**) Densitometry values for proteins that are induced upon ATRX depletion in CT2A cell lines shown in Fig. 3b and two other independent experiments (n=3). Data are presented as mean ± SEM and are normalized to β-actin loading control and *Atrx*^{WT} for every sample. Asterisks denote significant p-values from one-way ANOVA with Dunnett's post hoc test. (*: $p<0.05$; **: $p<0.01$; ***: $p<0.001$). (**e**) Densitometry values for proteins that are induced upon ATRX depletion in M059J cell lines shown in Supplementary Fig. 5c and two other independent experiments (n=3). Data are presented as mean ± SEM and are normalized to β-actin loading control and *ATRX*^{WT} for every sample. Asterisks denote significant p-values from unpaired, two-tailed Student's t-test (*: $p<0.05$; **: $p<0.01$; ***: $p<0.001$). Source data are provided as a Source Data file.

Supplementary figure 6

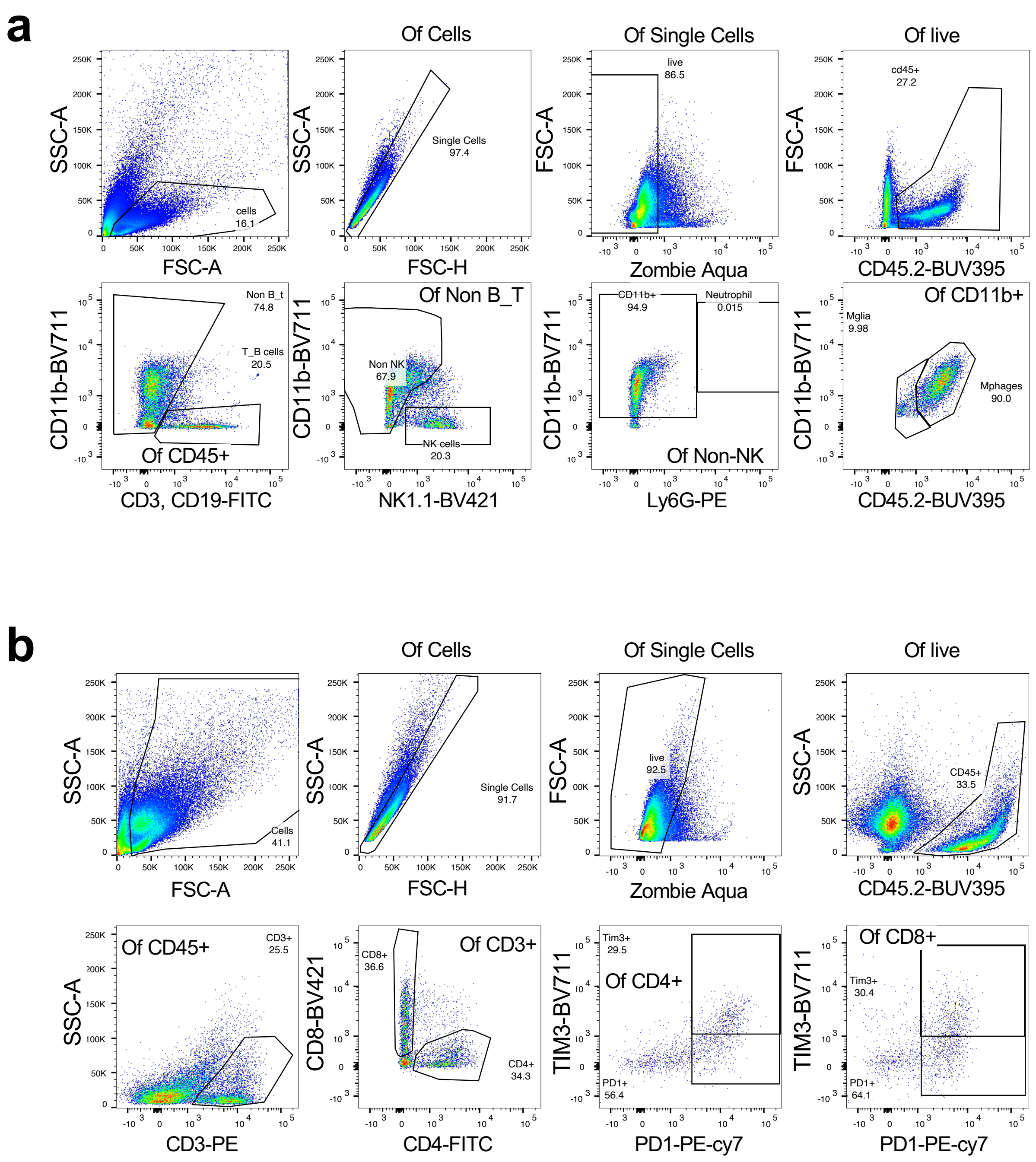

Supplementary figure 6 – supporting figure 4a. Representative gating strategy for analysis of *Atrx* KO tumors.

Gating strategy for analysis of tumor associated myeloid (**a**) and T cells (**b**) along with relevant phenotypes by flow cytometry.

Supplementary figure 7

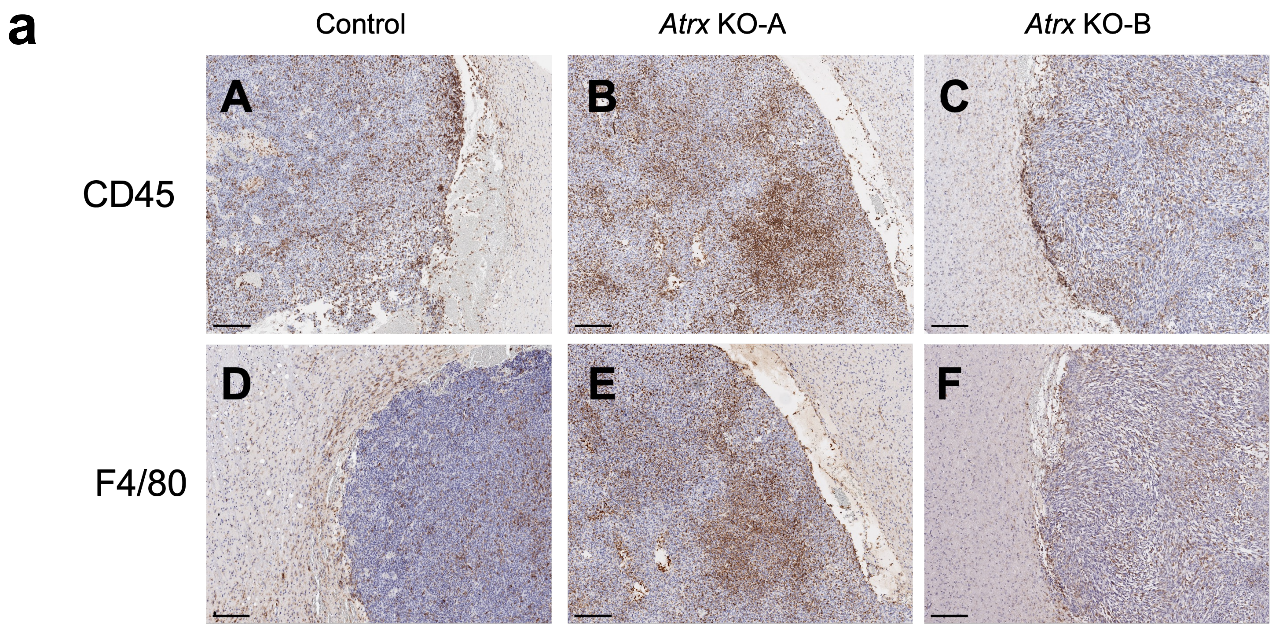

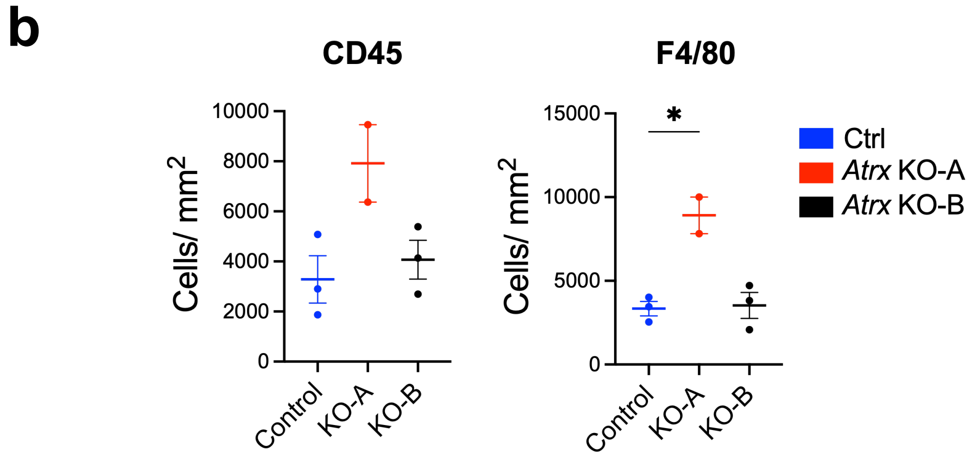

Supplementary figure 7 – supporting figure 4b. Immune infiltration in CT2A *Atrx*-KO tumors.
(a) Representative low magnification IHC images for CD45 (A, B, C) and F4/80 (D, E, F) expression (brown) in CT2A CRISPR Control (*Atrx*^{WT}) (A, D), *Atrx* KO-A (B, E) and KO-B (C, F) tumors, with hematoxylin counter-staining (blue). Scale bar: 200µm. Number of brains subjected to IHC per group: CRISPR Control – n=3; *Atrx* KO-A – n=2; *Atrx* KO-B – n=3. (b) Quantitation of CD45 and F4/80 expression in CT2A CRISPR control and *Atrx* KO tumors (N: Control – 3, *Atrx* KO-A – 2, *Atrx* KO-B - 3). Data are presented as mean ± SEM. Asterisks denote significant p-values from unpaired two-tailed Student's t-test (*: $p<0.05$). Source data are provided as a Source Data file.

Supplementary figure 8

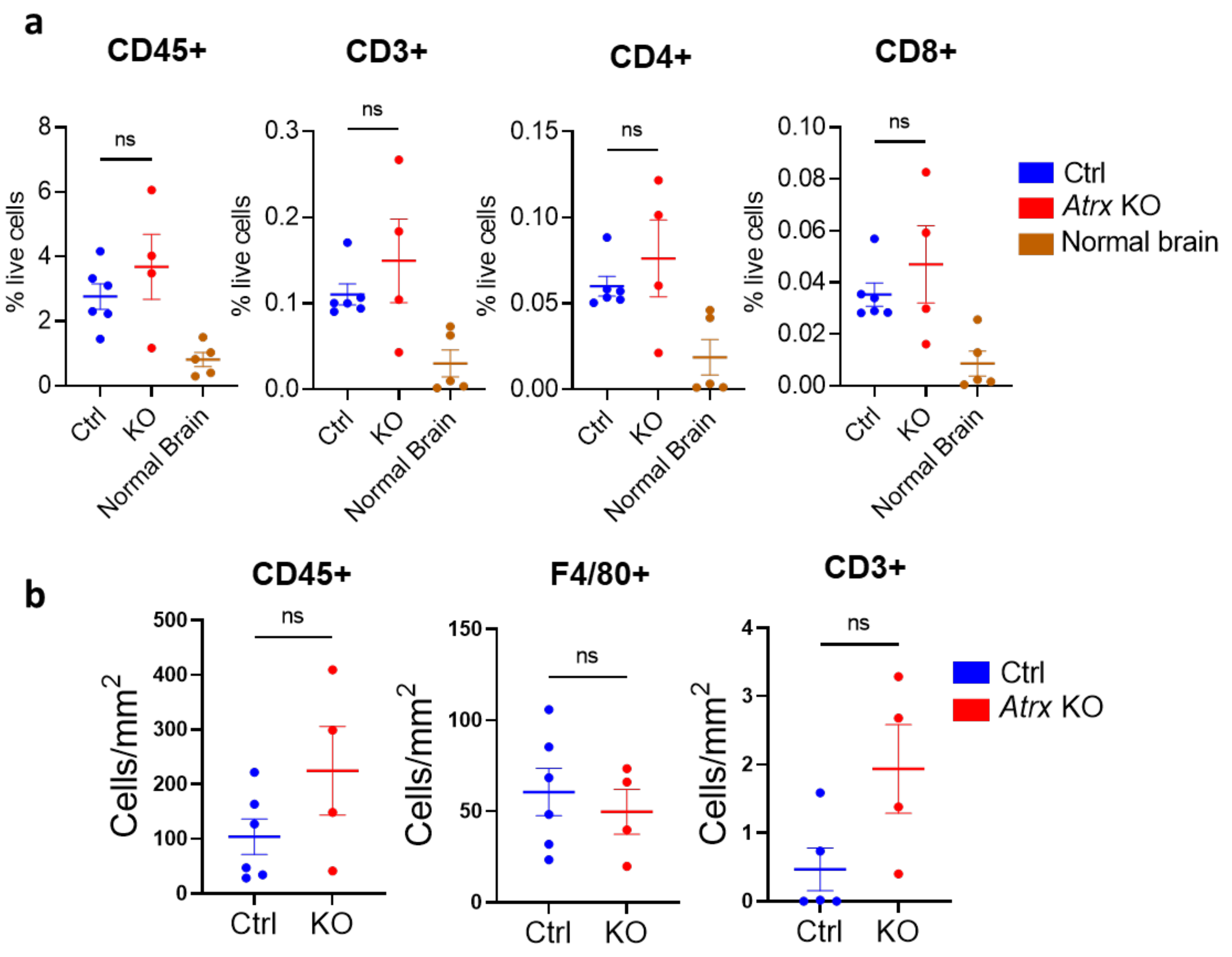

Supplementary figure 8 – supporting figure 4c. ATRX deficiency leads to increased immune cell infiltration.
(a) Flow cytometry analysis of RCAS/Ntv-a control (*Atrx*^{WT}) and *Atrx* KO tumor-bearing hemispheres harvested 14 days post-intracranial implantation, showing percent live cell density of CD45+ leukocytes, CD3+, CD4+, and CD8+ T-cells. Gating strategy is provided in Supplementary Fig. 6. n=6 for control; n=4 for *Atrx* KO; n=5 for normal brain. Data are presented as mean ± SEM. Statistics are the result of an unpaired two-tailed Student's t-test comparing control and *Atrx* KO samples. (b) Blinded quantitation of IHC staining for CD45, F4/80 and CD3 staining is displayed. n=6 for control; n=4 for *Atrx* KO. Data are presented as mean ± SEM. Statistics are the result of an unpaired two-tailed Student's t-test comparing control and *Atrx* KO samples. Source data are provided as a Source Data file.

Supplementary figure 9

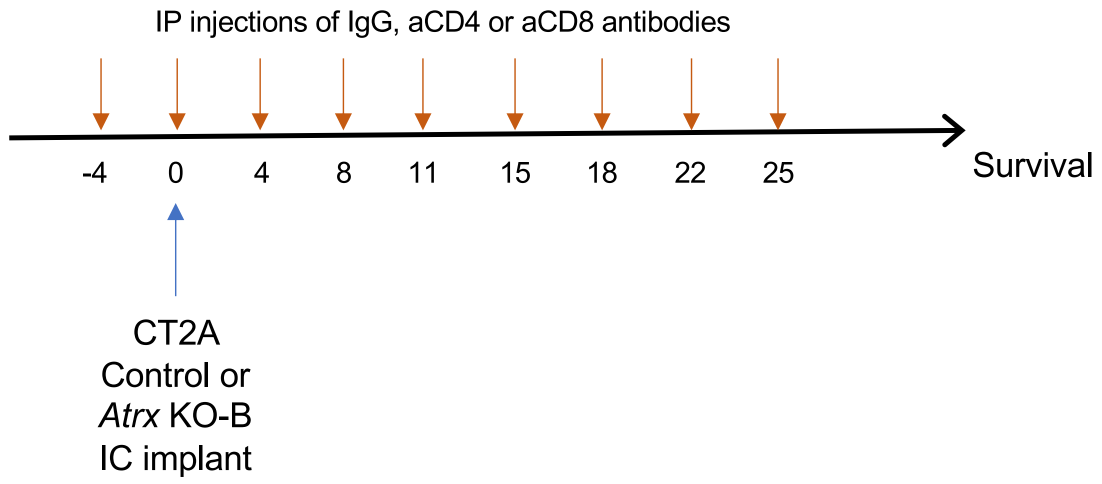

Supplementary Figure 9 – supporting figures 4d-e: Schema showing timeline of intraperitoneal (IP) injections of isotype control IgG, anti-CD4 and anti-CD8 antibodies in C57BL/6 mice bearing CT2A CRISPR control or *Atrx* KO-B tumors. Mice were dosed every 3 or 4 days till they exhibited neurological symptoms or loss in body weight, at which point they were sacrificed.

Supplementary figure 10

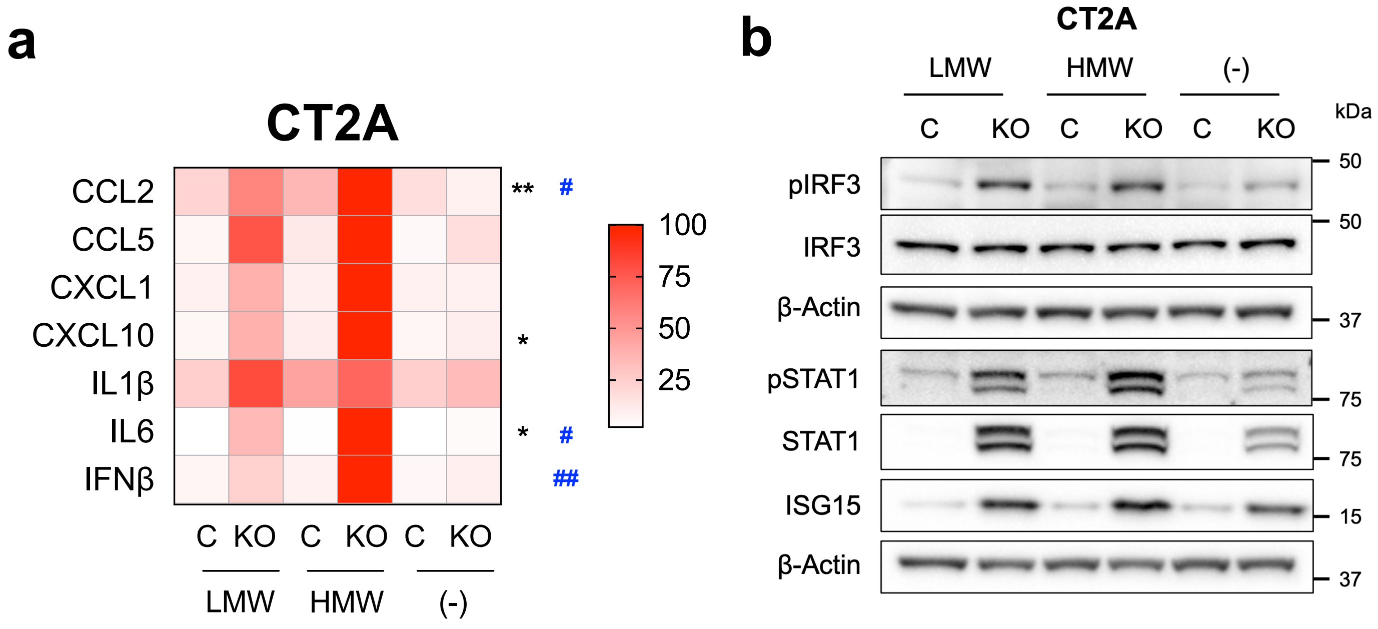

Supplementary figure 10 – supporting figures 6a and 6d. ATRX depletion sensitizes cells to poly(I:C), a dsRNA agonist.
(**a**) Cytokine levels in conditioned media from CT2A CRISPR control and *Atrx* KO-B cells, treated with 10µg/ml poly(I:C) LMW or HMW for 24hrs. Supernatant cytokines were analyzed by cytokine bead arrays for antiviral and proinflammatory cytokines. For heatmap generation, maximum values for each cytokine were set to 100%. Only cytokines and signaling proteins with observed induction after treatment with poly(I:C) are included. N=3 independent experiments. Asterisks & hashtags denote significant one-way ANOVA with Sidak's post-hoc test comparing both poly(I:C) LMW (*) and poly(I:C) HMW (#) between *Atrx*-KO and CRISPR control cell lines (*, #: p<0.05; **, ##: p<0.01; ***, ###: p<0.0001). Only cytokines and signaling proteins with observed induction after treatment with poly(I:C) are included.
(**b**) Representative Western blots using lysates from CT2A CRISPR control and *Atrx* KO-B cells treated with 10µg/ml poly(I:C) LMW or HMW for 4hrs or 24hrs, screened for pIRF3/IRF3, pSTAT1/STAT1 and ISG15 involved in innate immune signaling. β-actin serves as the loading control. N=3 independent experiments. Source data are provided as a Source Data file.

Supplementary figure 11

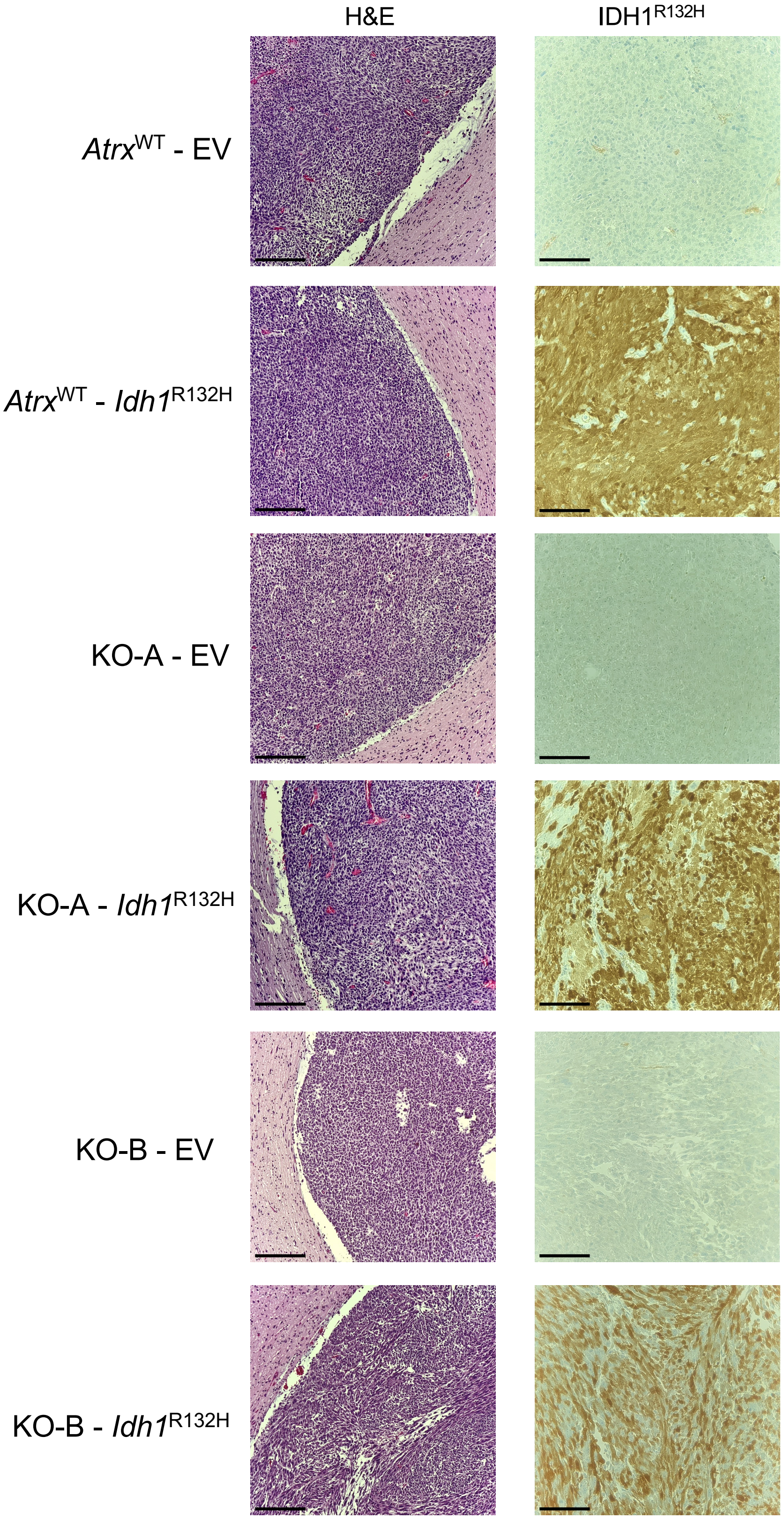

Supplementary figure 11 – supporting figure 7d. H&E and IDH1^{R132H} staining of CT2A *Idh1*^{WT} or *Idh1*^{R132H} tumors harvested when mice exhibited neurological symptoms or were moribund. H&E images were taken using a 10X objective (Scale bar: 200µm), while IDH1^{R132H} IHC images were taken using a 20X objective (Scale bar: 100µm).

Supplementary figure 12

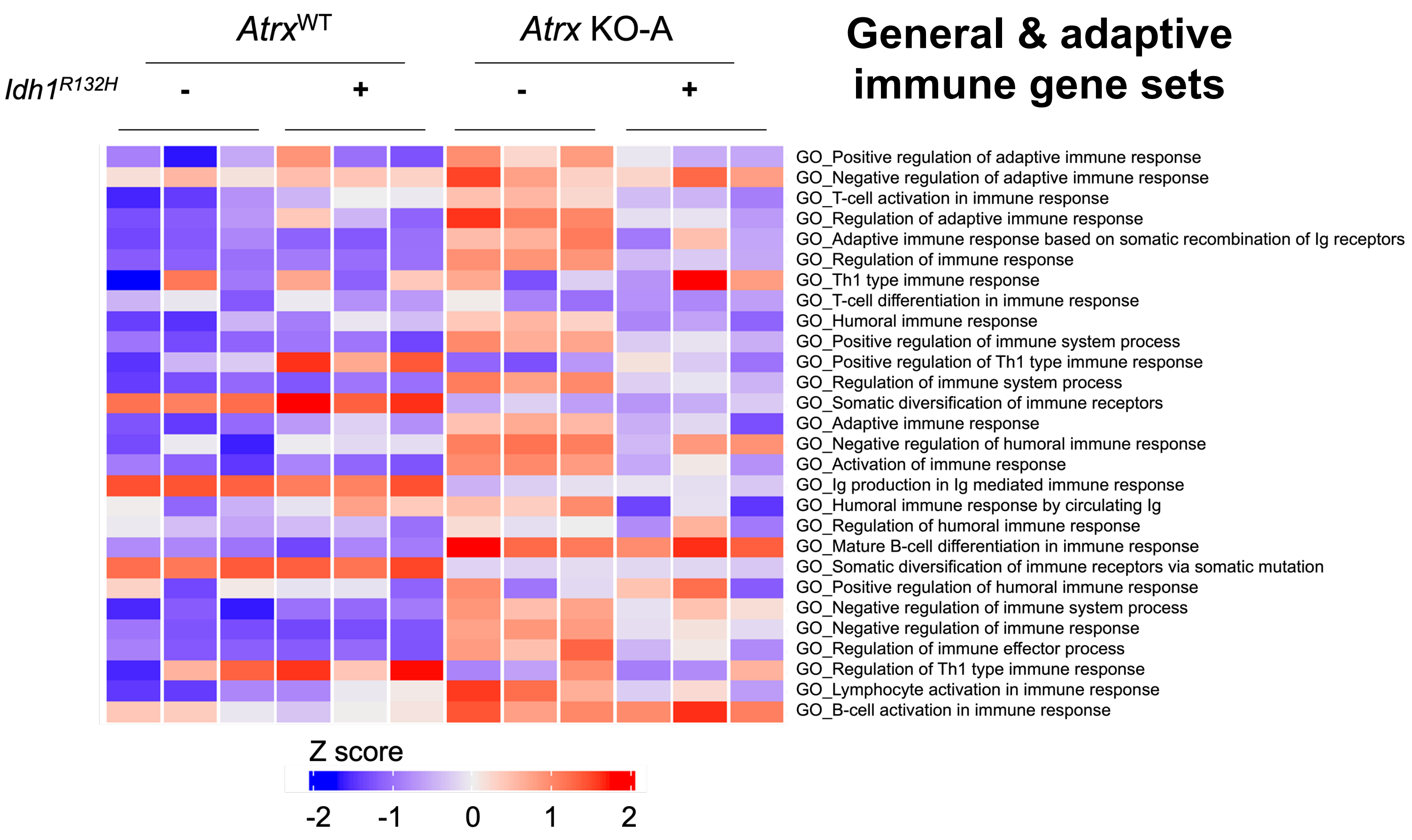

Supplementary figure 12 – supporting figure 8a. Idh1^{R132H} co-expression in Atrx-deficient KO-A cells dampens baseline innate gene expression.

Heatmap from ssGSEA showing loss of enrichment of various general and adaptive immune-related GO terms in *Atrx* KO-A/ *Idh1^{R132H}* cells compared to *Atrx KO*-A/ *Idh1^{WT}* cells. RNA was isolated from cells cultured for 72hrs. N=3 technical replicates per cell line.

Supplementary figure 13

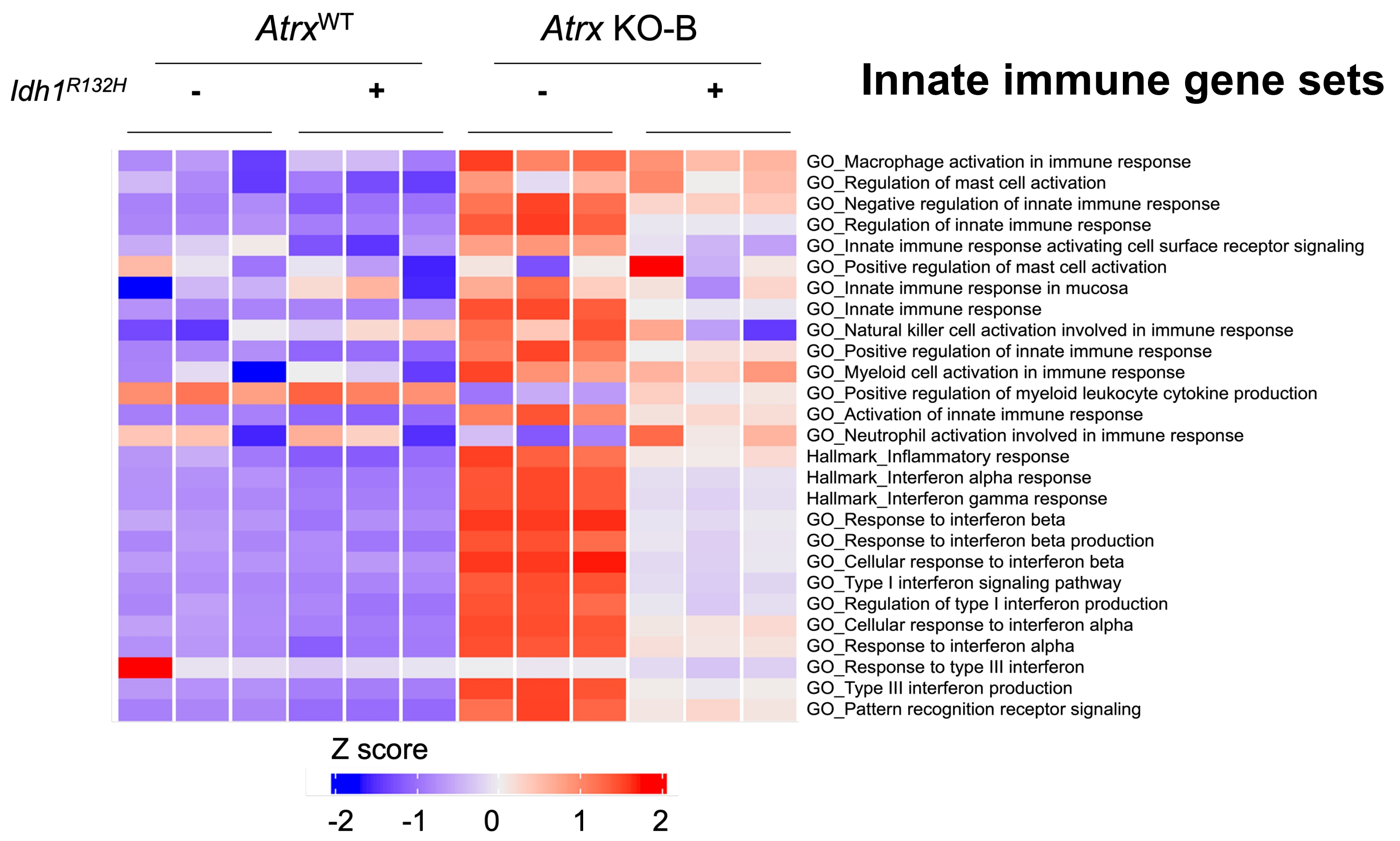

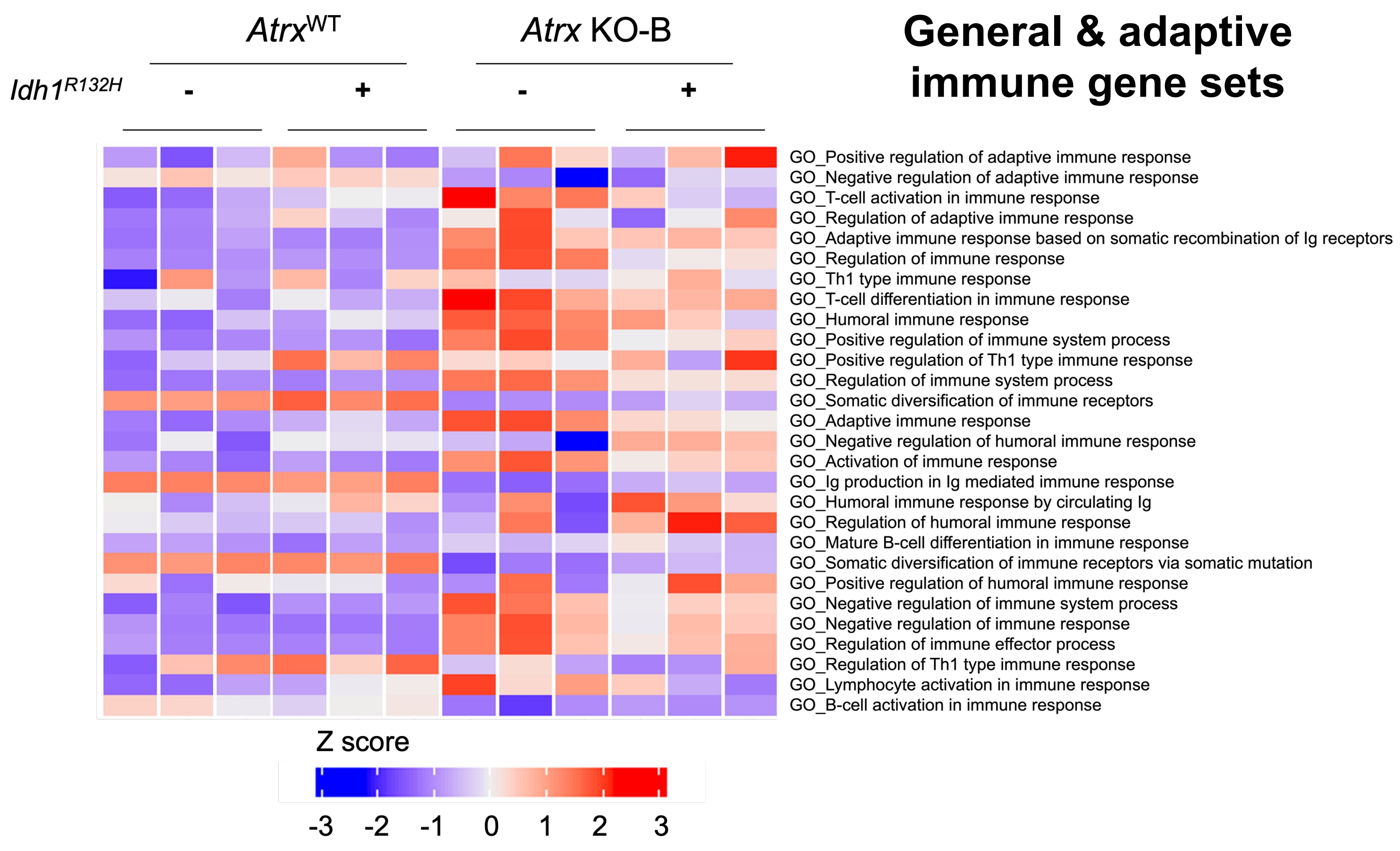

Supplementary figure 13 – supporting figure 8a. Idh1^{R132H} co-expression in Atrx-deficient KO-B cells dampens baseline innate gene expression.

Heatmap from ssGSEA showing loss of enrichment of various innate and adaptive immune-related GO terms in *Atrx* KO-B/ *Idh1*^{R132H} cells compared to *Atrx KO*-B/ *Idh1*^{WT} cells. RNA was isolated from cells cultured for 72hrs. N=3 technical replicates per cell line.

Supplementary figure 14

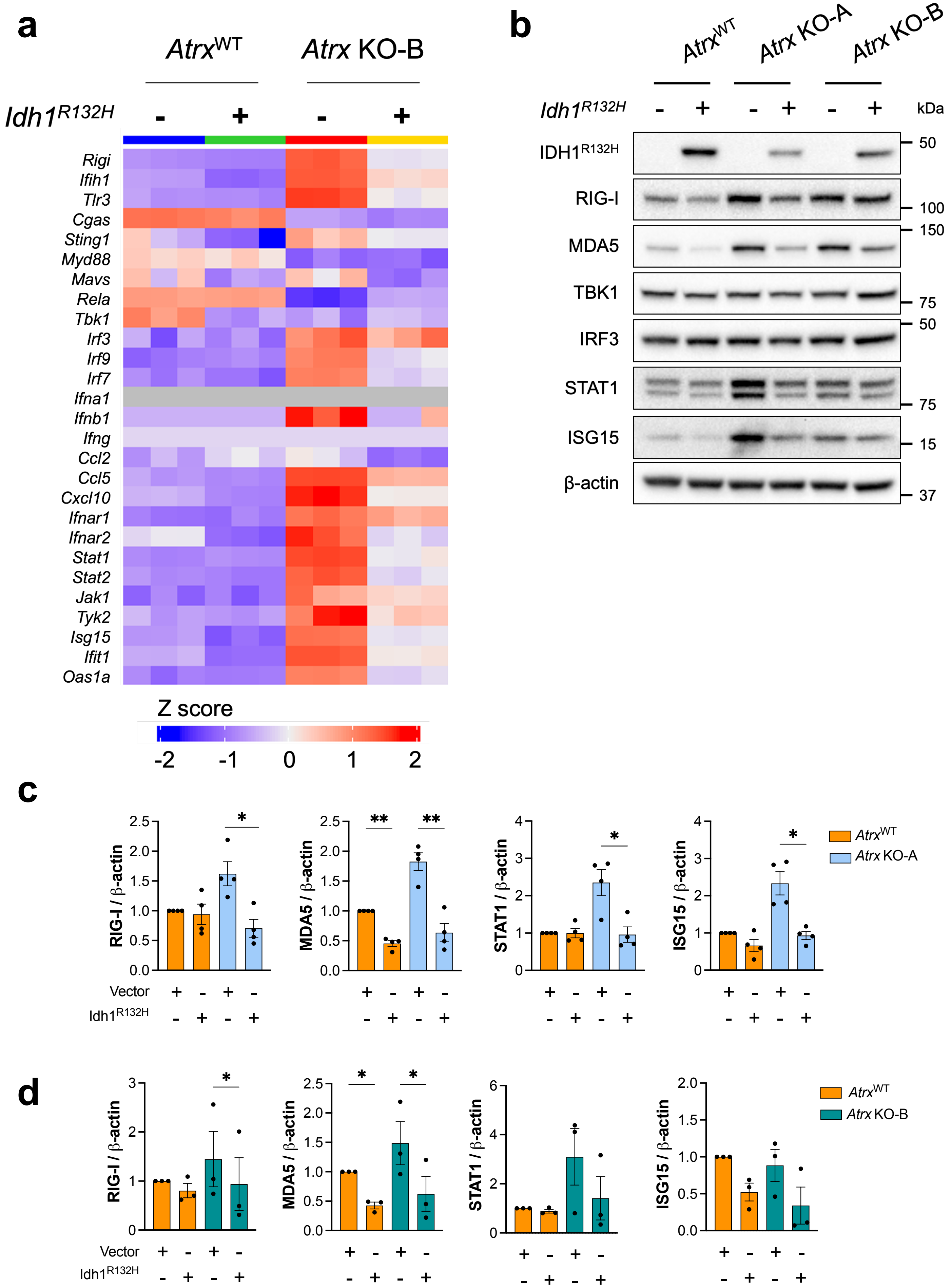

e

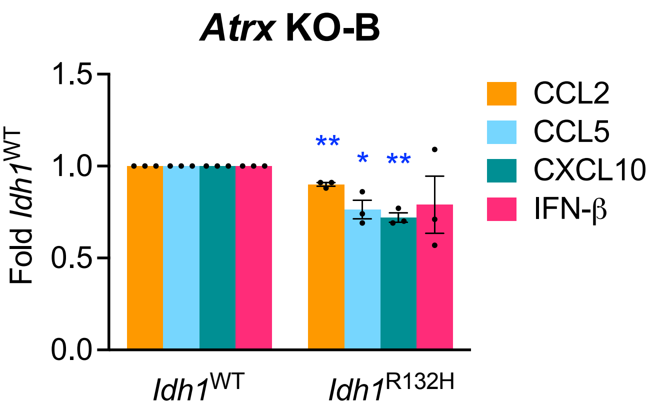

Supplementary figure 14 – supporting figures 8b-d. IDH1^{R132H} co-expression in ATRX-deficient cells dampens baseline innate protein expression and cytokine secretion.

(**a**) Heatmap showing differential expression of immune-related genes in CT2A *Atrx*^{WT} and *Atrx* KO-B cells with or without *Idh1*^{R132H}. n=3 technical replicates per cell line. (**b**) Lysates from CT2A CRISPR control (*Atrx*^{WT}), *Atrx* KO-A and *Atrx* KO-B cells expressing exogenous IDH1^{R132H,} or empty vector cultured for 72hrs, were screened by Western blotting for various innate immune proteins. b -actin serves as the loading control. Representative blots are shown; N=3 independent experiments. Densitometry values are indicated in Supplementary Fig 14d. (**c**) Densitometry values for proteins that are modulated upon IDH1^{R132H} co-expression in CT2A lines shown in Fig. 8c and three other independent experiments (n=4). Data are presented as mean $\pm$ SEM. (**d**) Densitometry values for proteins that are modulated upon IDH1^{R132H} co-expression in CT2A lines shown in Supplementary Fig. 14b and two other independent experiments (n=3). Data are presented as mean $\pm$ SEM and are normalized to β-actin loading control and *Atrx*^{WT}/ empty vector (*Idh1*^{WT}) for every sample. Asterisks in S14c and S14d denote significant p-values from one-way ANOVA with Sidak's post hoc test. (*: p<0.05; **: p<0.01; ***: p<0.001). (**e**) Conditioned media from CT2A *Atrx* KO-B cells expressing exogenous IDH1^{R132H} or empty vector cultured for 72hrs was assayed for antiviral and proinflammatory cytokines using a Legendplex assay kit. Data indicates fold change values normalized to corresponding *Idh1*^{WT} sample, shown as mean $\pm$ SEM. N=3 independent experiments. Asterisks denote significant results from unpaired two-tailed Student's t-tests. (*: p<0.05; **: p<0.01; ***: p<0.001). Source data are provided as a Source Data file.

Supplementary figure 15

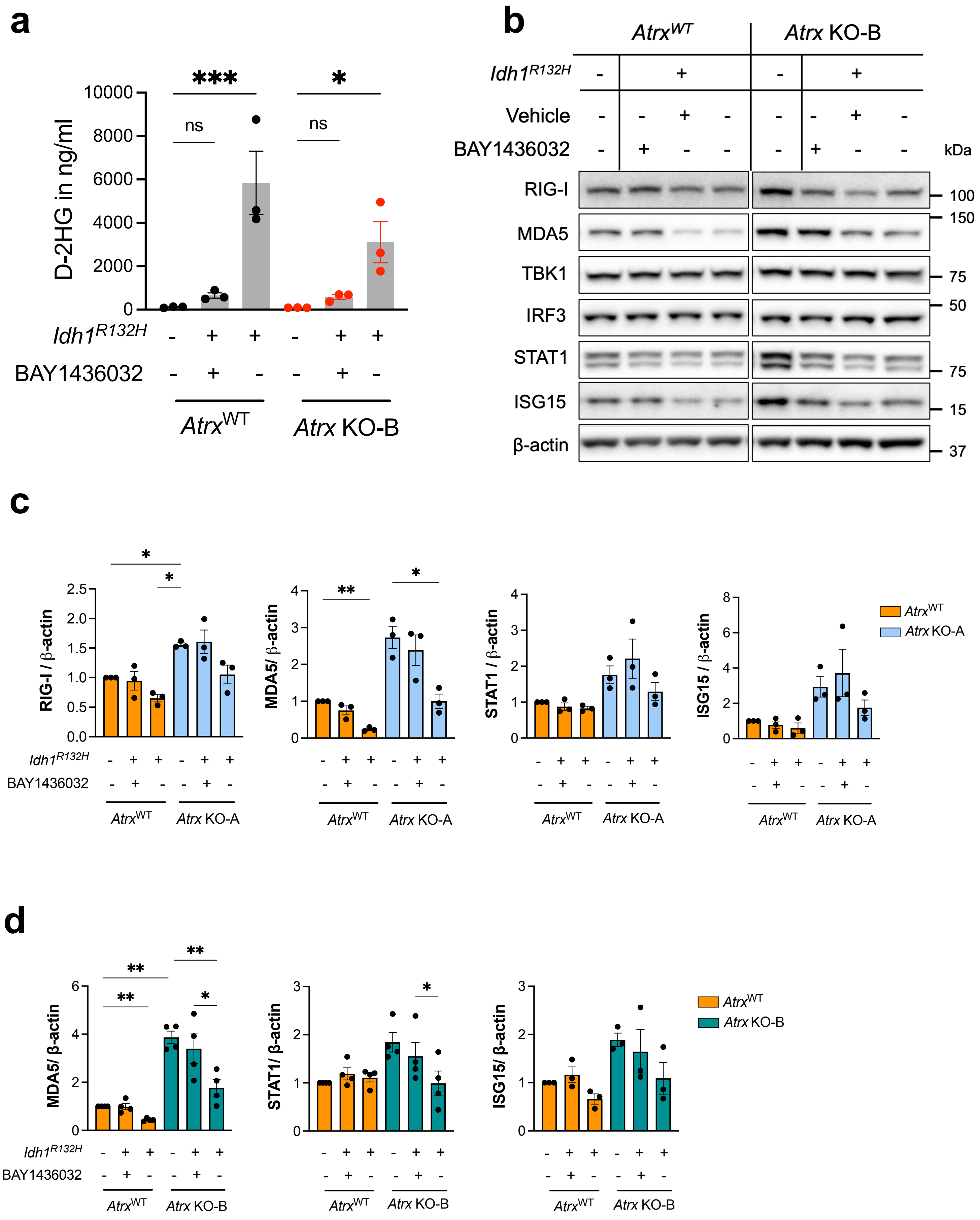

Supplementary figure 15 – supporting figure 9. BAY1436032 partially reverses *IDH1*^{R132H}-mediated immunosuppression.

(**a**) D-2HG levels in conditioned media from CT2A CRISPR control (*Atrx*^{WT}) or *Atrx* KO-B cells expressing IDH1^{R132H}, or empty vector treated with 1μM BAY1436032 or vehicle every day for 3 days, normalized to total protein. n=3 independent experiments. Data are presented as mean ± SEM. Asterisks indicate significant p-values from one-way ANOVA with Sidak post-hoc test. (*: p<0.05, **: p<0.01 ***: p<0.001). (**b**) Representative Western blots using lysates from CT2A CRISPR control (*Atrx*^{WT}) or *Atrx* KO-B cells expressing IDH1^{R132H} or empty vector that were treated with 1uM BAY1436032 or vehicle every day for 3 days, screened for proteins involved in innate immune signaling. β-actin serves as the loading control. N=3 independent experiments. (**c**) Densitometry values for proteins that are modulated upon BAY1436032 treatment in IDH1^{R132H} - expressing CT2A lines shown in Fig. 9b and two other independent experiments (n=3). Data are presented as mean ± SEM. (**d**) Densitometry values for proteins that are modulated upon BAY1436032 treatment in IDH1^{R132H} - expressing CT2A lines shown in Supplementary Fig. 15b and other independent experiments (n=4 for MDA5, STAT1 and n=3 for ISG15). Data are presented as mean ± SEM and are normalized to β-actin loading control and *Atrx*^{WT}/ EV + Vehicle for every sample. Asterisks in S15c and S15d denote significant p-values from one-way ANOVA with Tukey's post hoc test. (*: p<0.05; **: p<0.01; ***: p<0.001). Source data are provided as a Source Data file.

Supplementary figure 16

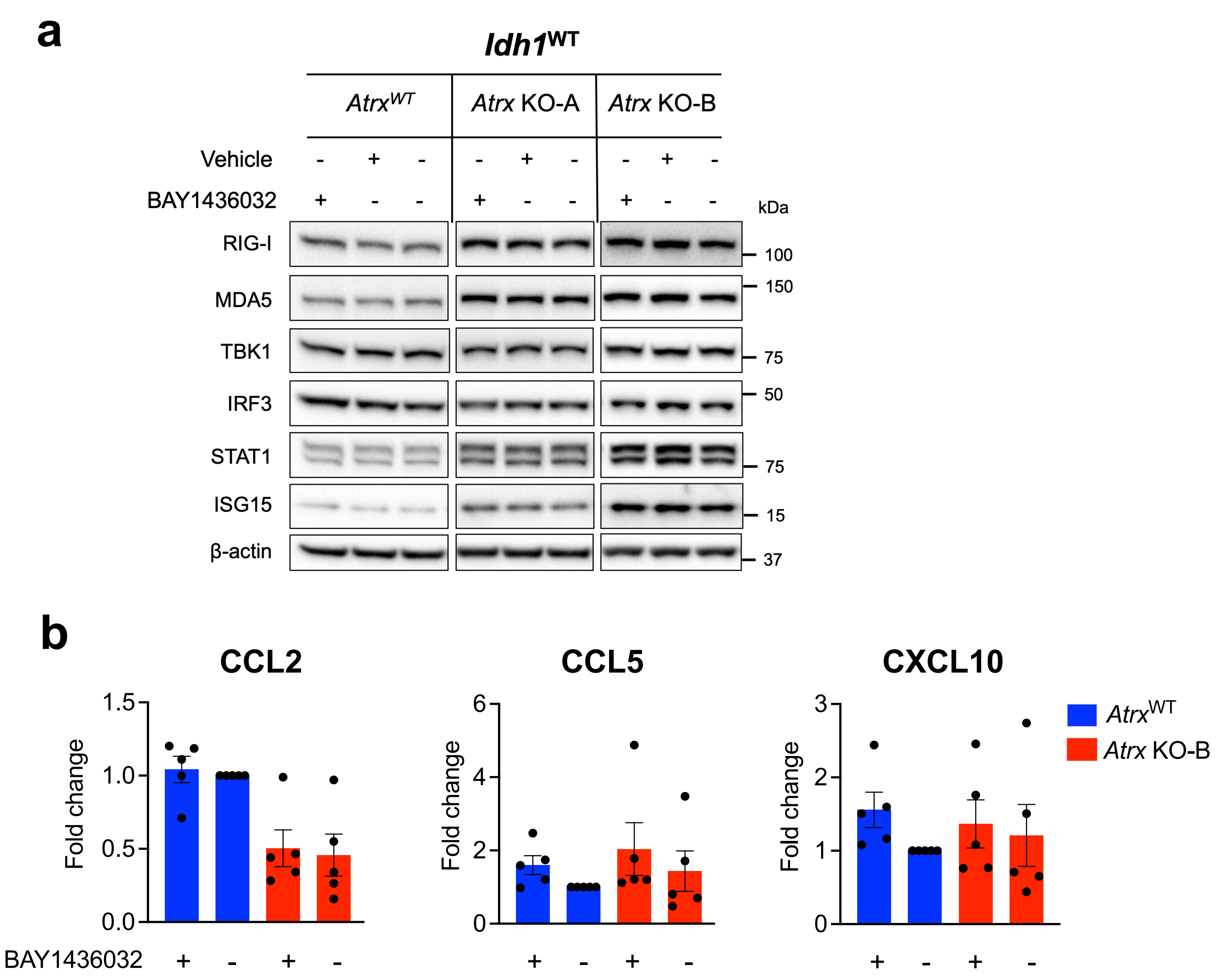

Supplementary figure 16 – supporting figure 9. BAY1436032 partially reverses *IDH1^{R132H}*-mediated immunosuppression.

(**a**) Representative Western blots using lysates from CT2A CRISPR control (*Atrx*^{WT}), *Atrx* KO-A or *Atrx* KO-B cells expressing MSCV empty vector (*Idh1*^{WT}) that were treated with 1µM BAY1436032 or vehicle every day for 3 days, screened for proteins involved in innate immune signaling. β-actin serves as the loading control. N=3 independent experiments. (**b**) Cytokine/ chemokine levels in conditioned media from CT2A CRISPR control (*Atrx*^{WT}) and *Atrx* KO-B cells expressing IDH1^{R132H} treated with 1µM BAY1436032 or vehicle every day for 3 days. Supernatant cytokines were analyzed by cytokine bead arrays for antiviral and proinflammatory cytokines. Fold change values normalized to vehicle treated *Atrx*^{WT} sample are plotted. Data are presented as mean $\pm$ SEM. N=3 independent experiments . One-way ANOVA with Tukey's post-hoc test did not reveal any significant differences between groups. Source data are provided as a Source Data file.

Supplementary figure 17

a

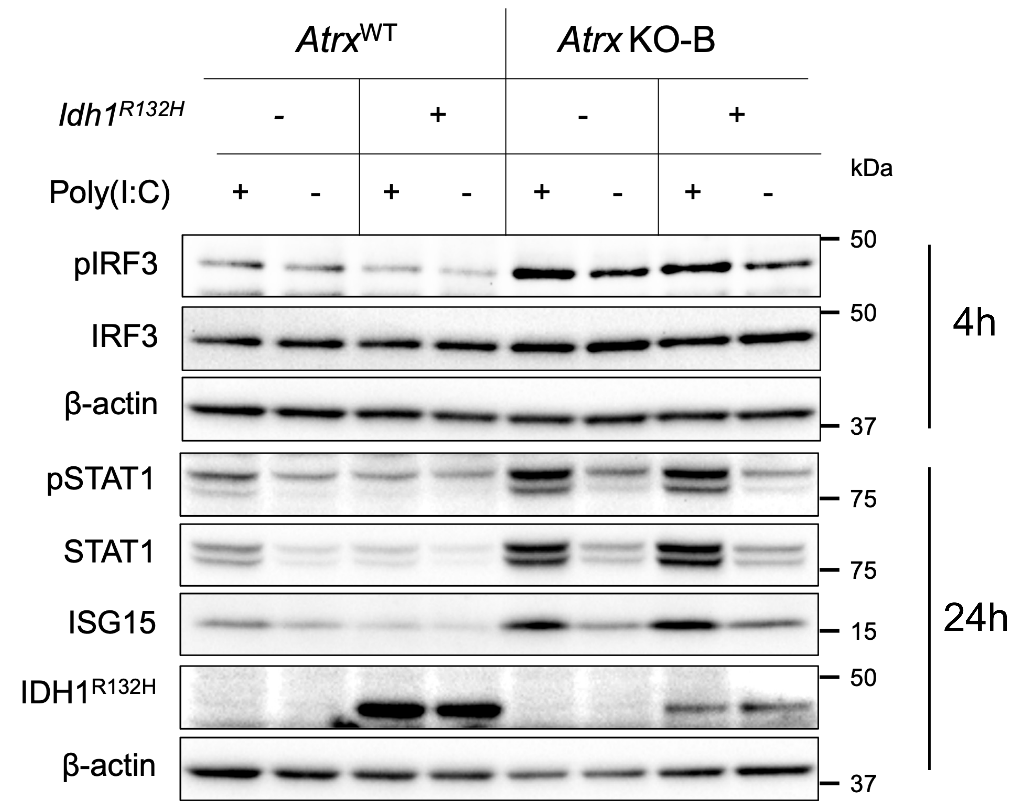

b

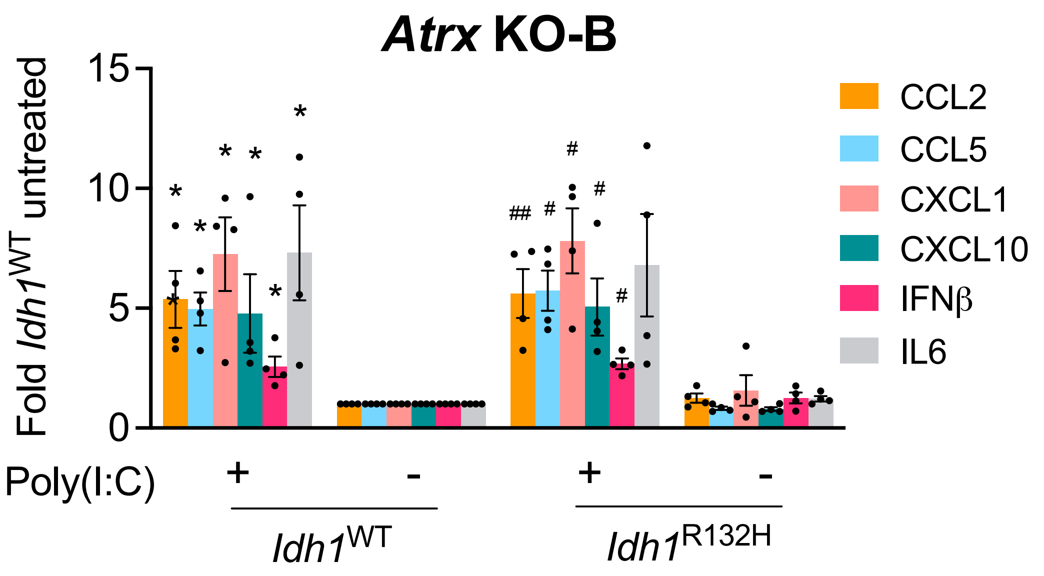

Supplementary figure 17 - supporting main figure 10a-b. *Atrx* KO/ *Idh1^{R132H}* cells retain sensitivity to poly(I:C).

(**a**) Representative Western blots using lysates from CT2A CRISPR control (*Atrx*^{WT}) or *Atrx* KO-B cells expressing IDH1^{R132H} or empty vector that were treated with 10µg/ml poly(I:C) HMW for 4hrs or 24hrs and screened for proteins involved in innate immune signaling. N=3 independent experiments. (**b)** Cytokine levels in conditioned media from CT2A *Atrx* KO-B cells expressing Idh1^{R132H} or empty vector treated with poly(I:C) HMW for 24hrs. Supernatant cytokines were analyzed by cytokine bead arrays for antiviral and proinflammatory cytokines. Fold change values normalized to untreated *Atrx* KO-B/ *Idh1*^{WT} sample are shown as mean $\pm$ SEM. n= 4 independent experiments. Asterisks indicate significant p-values from one-way ANOVA with Tukey's post hoc test. Source data are provided as a Source Data file.

Supplementary table 1: Sequences of sgRNAs targeting human and mouse ATRX.

sgRNA-hATRX-e9-sense	CACCGTGTTGGCAGGTTCATATTG
sgRNA-hATRX-e9-antisense	AAACCAATATGAACCTGCCAACAC

sgRNA-mATRX-ex9-1-sense	CACCGTGTAAAAACTACACCGTTG
sgRNA-mATRX-ex9-1-antisense	AAACCAACGGTGTAGTTTTTACAC
sgRNA-mATRX-ex9-2-sense	CACCGTATCTGACGATGAACACTC
sgRNA-mATRX-ex9-2-sense	AAACGAGTGTTCATCGTCAGATAC

Supplementary table 2: Antibodies used in this study.

Application	Antibody	Clone number	Company	Catalog number	Dilution
Western Blot	ATRX (Human specific)	D1N2E	Cell signaling	14820	1:1000
	ATRX	-	Novus Biologicals	NBP1-32851	1:1000
	ATRX	E5X7O	Cell signaling	10321	1:500
	$IDH1^{R132H}$	H09	Dianova	DIA-H09	1:1000
	$IDH1^{WT}$	D2H1	Cell signaling	8137	1:1000
	Phospho IRF3 (Ser396)	4D4G	Cell signaling	4947	1:1000
	Phospho IRF3 (Ser396)	E.875.8	Thermo scientific	MA5-14947	1:1000
	Total IRF3 (Human specific)	D9J5Q	Cell signaling	10949	1:1000
	Total IRF3	12A4A35	Biolegend	655702	1:1000
	Phospho STAT1 (Tyr701)	58D6	Cell signaling	9167	1:1000
	Total STAT1	-	Cell signaling	9172	1:2000
	Total STAT1	D1K9Y	Cell signaling	14994	1:1000
	Total TBK1	D1B4	Cell signaling	3504	1:1000
	ISG15	-	Cell signaling	2743	1:2000
	RIG-I	D14G6	Cell signaling	3743	1:1000
	MDA5	D74E4	Cell signaling	5321	1:1000
	Vinculin	hVIN-1	Sigma	V9264	1:5000
	β-actin	-	Cell signaling	4967	1:5000
	β-actin, HRP conjugated	13E5	Cell signaling	5125	1:5000
	β-tubulin, HRP conjugated	9F3	Cell signaling	5346	1:5000
	Anti-rabbit IgG, HRP conjugated antibody	-	Cell signaling	7074	1:2000-1:5000
	Anti-mouse IgG, HRP conjugated antibody	-	Cell signaling	7076	1:2000
IHC	ATRX	E5X7O	Cell signaling	10321	1:200
	CD3	E4T1B	Cell Signaling	78588	1:100
	CD45	-	Abcam	ab10558	1:2000
	F4/80	BM8	Thermo scientific	14-4801-82	1:200
	$IDH1^{R132H}$	MRQ-67	Cell Marque (Sigma)	456R-34	1:25
Flow cytometry	CD45.2-BUV395	104	BD Biosciences	564616	1:100
	CD45-BUV395	30-F11	BD Biosciences	564279	1:100
	CD3-FITC	17A2	BioLegend	100204	1:100

	CD3-PE	17A2	Biolegend	100205	1:100
	CD19-FITC	1D3/CD19	BioLegend	152404	1:100
	NK1.1-BV421	PK136	BioLegend	108732	1:100
	NK1.1-BV605	PK136	Biolegend	108753	1:100
	CD11b-BV711	M1/70	BioLegend	101242	1:100
	CD11b-APC-Cy7	ICRF44	BD Biosciences	560914	1:100
	CD4-FITC	RM4-5	BioLegend	100510	1:100
	CD4-FITC	GK1.5	Biolegend	100406	1:100
	CD8-BV421	53-6.7	BioLegend	100738	1:100
	F4/80-APC	BM8	Invitrogen	17-4801-82	1:100
	Ly6G-PE	1A8	BioLegend	127608	1:100
	Ly-6G- PE-Cy7	1A8	BD Biosciences	560601	1:100
	Ly-6C-PerCP/Cy5.5	HK1.4	Biolegend	128012	1:100
	IA/IE-BV786	M5/114.15.2	BD Biosciences	742894	1:100
T-cell depletion	*InVivo*MAb Rat IgG2b Isotype control	LTF-2	BioXcell	BE0090	250μg per mouse
	*InVivo*MAb Anti-mouse CD4	GK1.5	BioXcell	BE0003-1	250μg per mouse
	*InVivo*MAb Anti-mouse CD8α	2.43	BioXcell	BE0061	250μg per mouse
