# Supplementary material for: Interplay between ATRX and IDH1 mutations governs innate immune responses in diffuse gliomas
Source: Nat Commun. 2024 Jan 25;15:730. doi: 10.1038/s41467-024-44932-w (PMC10810843; doi:10.1038/s41467-024-44932-w)
Supplement: Supplementary file 2 — Reporting Summary [file 41467_2024_44932_MOESM2_ESM.pdf]

nature portfolio

Corresponding author(s): David M. Ashley, Jason T. Huse

Last updated by author(s): Dec 6, 2023

Reporting Summary

Nature Portfolio wishes to improve the reproducibility of the work that we publish. This form provides structure for consistency and transparency in reporting. For further information on Nature Portfolio policies, see our Editorial Policies and the Editorial Policy Checklist.

Statistics

For all statistical analyses, confirm that the following items are present in the figure legend, table legend, main text, or Methods section.

n/a	Confirmed	
☐	☒	The exact sample size (*n*) for each experimental group/condition, given as a discrete number and unit of measurement
☐	☒	A statement on whether measurements were taken from distinct samples or whether the same sample was measured repeatedly
☐	☒	The statistical test(s) used AND whether they are one- or two-sided<br>*Only common tests should be described solely by name; describe more complex techniques in the Methods section.*
☐	☒	A description of all covariates tested
☐	☒	A description of any assumptions or corrections, such as tests of normality and adjustment for multiple comparisons
☐	☒	A full description of the statistical parameters including central tendency (e.g. means) or other basic estimates (e.g. regression coefficient) AND variation (e.g. standard deviation) or associated estimates of uncertainty (e.g. confidence intervals)
☐	☒	For null hypothesis testing, the test statistic (e.g. *F*, *t*, *r*) with confidence intervals, effect sizes, degrees of freedom and *P* value noted<br>*Give P values as exact values whenever suitable.*
☒	☐	For Bayesian analysis, information on the choice of priors and Markov chain Monte Carlo settings
☒	☐	For hierarchical and complex designs, identification of the appropriate level for tests and full reporting of outcomes
☒	☐	Estimates of effect sizes (e.g. Cohen's *d*, Pearson's *r*), indicating how they were calculated

Our web collection on statistics for biologists contains articles on many of the points above.

Software and code

Policy information about availability of computer code

Data collection	Illumina HiSeq 4000 and NovaSeq 6000 S-prime flowcell were used for mRNA sequencing.<br>Agilent 1200 series HPLC and Sciex/Applied Biosystems API 5500 QTrap were used to measure D2HG levels in conditioned media and cell pellets using Analyst 1.6.2 software.<br>Image Lab 6.1 was used to collect Western blot images from a Biorad Chemidoc MP imaging system. Tecan i-control 2.0 was used to collect absorbance values from a Tecan Infinite M200 Pro plate reader.<br>BD FACS Diva software was used to collect flow cytometry data from a Fortessa X20.<br><br>Echo Pro software was used to acquire bright field images from CT2A tumors using an Echo Revolve microscope. Bright field images for ATRX and CD3 IHC images for the RCAS tumors were collected with a Keyence BZ-X810 microscope, using the BZ-X800 viewer acquisition software. Leica Aperio AT2 slide scanner was used by Histowiz to visualize and acquire bright field images.
Data analysis	RNAseq data were processed and analyzed using HisSeq Control Software, Illumina bcl2fastq 2 software, FastQC v0.11.9, MultiQC v1.11 and Trimmomatic v.0.36. STAR aligner v.2.7.2b and v.2.7.8a were used to map sequence to either mouse or human genome. Quantification and generation of the raw counts matrix was performed via featureCounts v1.6.3 or via HTseq-count tool v.0.9.1.<br>Differential expression calculation was performed within RStudio 2022.07.0 Build 548 running R version 4.2.1. , using DeSeq2 R package v1.38.2 or limma-voom 3.44.3. Single sample gene set enrichment analysis (ssGSEA) was performed using R package, GSVA v1.47.0 with method set to “ssgsea” and a list of custom gene sets. The desktop version of GSEA was also used. Heatmaps were generated via the package ComplexHeatmap v2.14.0.<br>Publicly available processed single cell RNAseq data were extracted and analyzed using Seurat’s v4.3.0 standard pre-processing workflow with clustering resolution determination aided by the R package clustree v0.5.0. Differential expression was calculated using the FindAllMarkers

function. Differential expression results were input into the GSEA R package fgsea v1.22.0 along with the Hallmark gene set collection.
Analyst 1.6.2 software (Sciex) was used for D2HG data acquisition, integration of chromatograms, calibration curve calculation, and quantification of study samples.
Western blot images were quantified using Image Lab 6.1 software.
Legendplex Data Analysis Software suite available online (BioLegend) was used to calculate concentrations of cytokines in conditioned media.
Flow cytometry data were analyzed using Flow-Jo v10.8.1 (BD Biosciences).
IHC images were quantified using QuPath software (v0.4.4).
GraphPad Prism v9.5.1 was used to generate plots, cytokine heatmaps and perform statistical analysis.

For manuscripts utilizing custom algorithms or software that are central to the research but not yet described in published literature, software must be made available to editors and reviewers. We strongly encourage code deposition in a community repository (e.g. GitHub). See the Nature Portfolio guidelines for submitting code & software for further information.

Data

Policy information about availability of data

All manuscripts must include a data availability statement. This statement should provide the following information, where applicable:

- Accession codes, unique identifiers, or web links for publicly available datasets
- A description of any restrictions on data availability
- For clinical datasets or third party data, please ensure that the statement adheres to our policy

All data generated during this study are included in this published article, supplementary data files or in the source data file. All transcriptional data generated in this study have been deposited in Gene Expression Omnibus (GEO) under accession numbers: GSE228242 (CT2A Atrx-KO cell lines) (https://www.ncbi.nlm.nih.gov/geo/query/acc.cgi?acc=GSE228242), GSE228181 (CT2A Atrx-KO/ Idh1 mutant cell lines) (https://www.ncbi.nlm.nih.gov/geo/query/acc.cgi?acc=GSE228181), GSE228243 (M059J ATRX-KO cell lines) (https://www.ncbi.nlm.nih.gov/geo/query/acc.cgi?acc=GSE228243), GSE231830 (RCAS Atrx-KO cell lines) (https://www.ncbi.nlm.nih.gov/geo/query/acc.cgi?acc=GSE231830) and GSE231831 (RCAS Atrx-KO tumors) (https://www.ncbi.nlm.nih.gov/geo/query/acc.cgi?acc=GSE231831).
The following publicly available datasets were also analyzed for this study - CNS/Brain TCGA LGG PanCancer Atlas Study from cBioportal (https://www.cbioportal.org/study/summary?id=lgg_tcga_pan_can_atlas_2018) and scRNA-Seq datasets from GEO - IDH-mutant astrocytoma (GSE89567) (https://www.ncbi.nlm.nih.gov/geo/query/acc.cgi?acc=GSE89567) and oligodendroglioma (GSE70630) (https://www.ncbi.nlm.nih.gov/geo/query/acc.cgi?acc=GSE70630).
Source data are provided with this paper.

Research involving human participants, their data, or biological material

Policy information about studies with human participants or human data. See also policy information about sex, gender (identity/presentation), and sexual orientation and race, ethnicity and racism.

Reporting on sex and gender	N/A
Reporting on race, ethnicity, or other socially relevant groupings	N/A
Population characteristics	N/A
Recruitment	N/A
Ethics oversight	N/A

Note that full information on the approval of the study protocol must also be provided in the manuscript.

Field-specific reporting

Please select the one below that is the best fit for your research. If you are not sure, read the appropriate sections before making your selection.

☒ Life sciences ☐ Behavioural & social sciences ☐ Ecological, evolutionary & environmental sciences

For a reference copy of the document with all sections, see nature.com/documents/nr-reporting-summary-flat.pdf

Life sciences study design

All studies must disclose on these points even when the disclosure is negative.

Sample size	Power analysis was not performed to determine sample sizes, rather prior experience and pilot studies informed sample size [1-2]. Sufficient number of replicates that will yield statistical power were chosen as mentioned in figures. Most of the in vitro experiments are representative of a minimum of three independent experiments, while for in vivo survival analysis, a minimum of 10 animals per group was chosen for statistical analysis. In vivo experiments were also independently repeated two times.<br>1. Brown, MC et al. Nature Communications, 2021, 12 :1858, doi.org/10.1038/s41467-021-22088-1<br>2. Danussi, C et al. Nature Communications, 2018, 13;9 :1057. doi: 10.1038/s41467-018-03476-6
Data exclusions	For tumor flow analysis (Fig 4a), two samples from CT2A Atrx KO-A group were excluded due to myelin contamination while processing, which confounded data collection and analysis. Otherwise, no other data was excluded from analyses.

Replication	Experiments designed to understand the role of ATRX loss (Figures 1-6; Supplementary figures 3-8) were reproducible across 3 different model systems - mouse CT2A model, human M059J model and RCAS/Nt-va model. Two different CT2A Atrx KO single cell clones, KO-A and KO-B were also compared side-by-side for all relevant experiments to ensure reproducibility. Most of the in vitro experiments were repeated at least 3 times as stated in each figure and all attempts at replication were successful. Details about biological or technical replicates are included for all figures. In vivo experiments and tumor flow cytometry analysis were independently repeated two times for every cell line. Individual data points are shown for all graphical analysis. For Western blot data, representative images are shown in figures and quantitation is shown for Westerns that show modest changes in protein expression . RNAseq analysis was performed on either 2 technical replicates (CT2A Atrx KO lines), 3 technical replicates (M059J ATRX KO lines, CT2A mutant IDH1 lines), 3 consecutive passages (RCAS Ntv-a cell lines) or 3 individual tumors (RCAS/ Ntv-a mouse tumors).
Randomization	In vitro experiments: Cells were randomly assigned to control or treatment groups and a minimum of three independent experiments or biological replicates were performed to ensure reporoducibility. In vivo work: Cages of mice were randomly assigned to different groups and equal distributions of starting weights for each group were confirmed.
Blinding	Investigators were blinded to group allocation during animal monitoring, flow cytometry data acquisition and analysis, Legendplex assay-based setup for cytokine measurements and analysis, D2HG measurements and tissue processing and staining. Blinding was not feasible for cell culture experiments, treatments and Western blotting because the investigator involved in allocating groups also performs treatments, sample collection, processing, data collection and analysis. Blinding was not necessary for analyzing gene expression (RNAseq) data.

Reporting for specific materials, systems and methods

We require information from authors about some types of materials, experimental systems and methods used in many studies. Here, indicate whether each material, system or method listed is relevant to your study. If you are not sure if a list item applies to your research, read the appropriate section before selecting a response.

Materials & experimental systems

n/a	Involved in the study
☐	☒ Antibodies
☐	☒ Eukaryotic cell lines
☒	☐ Palaeontology and archaeology
☐	☒ Animals and other organisms
☒	☐ Clinical data
☒	☐ Dual use research of concern
☒	☐ Plants

Methods

n/a	Involved in the study
☒	☐ ChIP-seq
☐	☒ Flow cytometry
☒	☐ MRI-based neuroimaging

Antibodies

Antibodies used	All antibodies used in this study are also listed in supplementary table 2 along with the respective catalog number,clone number and supplier name.<br>Antibodies for Western blotting: (Antibody, Clone number (if applicable), Cat no, Supplier)<br>Actin, 4967, Cell Signaling<br>ATRX (Human specific), Clone D1N2E, 14820, Cell Signaling<br>ATRX, NBP1-32851, Novus Biologicals<br>ATRX, Clone E5X7O, 10321, Cell Signaling<br>IDH1 R132H, Clone H09, DIA-H09, Dianova<br>IDH1, Clone D2H1, 8137, Cell signaling<br>Phospho IRF3 (Ser396), Clone 4D4G, 4947, Cell signaling<br>Phospho IRF3 (Ser396), Clone E.875.8, MA5-14947, Thermo scientific<br>Total IRF3 (Human specific), Clone D9J5Q, 10949, Cell signaling<br>Total IRF3, Clone 12A4A35, 655702, Biolegend<br>Phospho STAT1 (Tyr701), Clone 58D6, 9167, Cell signaling<br>STAT1, 9172, Cell signaling<br>STAT1, Clone D1K9Y, 14994, Cell signaling<br>TBK1, Clone D1B4, 3504, Cell signaling<br>ISG15, 2743, Cell signaling<br>RIG-I, Clone D14G6, 3743, Cell signaling<br>MDA5, Clone D74E4, 5321, Cell signaling<br>Vinculin, Clone hVIN-1, V9264, Sigma<br>HRP conjugated beta-actin, Clone 13E5, 5125, Cell signaling<br>HRP conjugated beta- tubulin, Clone 9F3, 5346, Cell signaling<br>Anti-rabbit IgG, HRP conjugated antibody, 7074, Cell signaling<br>Anti-mouse IgG, HRP conjugated antibody, 7076, Cell signaling<br><br>Antibodies for IHC: (Antibody, Clone number (if applicable), Cat no, Supplier)<br>ATRX, Clone E5X7O, 10321, Cell signaling

CD3, Clone E4T1B, 78588, Cell Signaling
CD45, ab10558, Abcam
F4/80, Clone BM8, 14-4801-82, Thermo scientific
IDH1 R132H, Clone MRQ-67, 456R-34, Cell Marque (Sigma)

Antibodies for flow cytometry: (Antibody, Clone number, Cat no, Supplier)
CD45.2-BUV395, Clone 104, 564616, BD Biosciences
CD45-BUV395, Clone 30-F11, 564279, BD Biosciences
CD3-FITC, Clone 17A2, 100204, BioLegend
CD3-PE, Clone 17A2, 100205, BioLegend
CD19-FITC, Clone 1D3/CD19, 152404, BioLegend
NK1.1-BV421, Clone PK136, 108732, BioLegend
NK1.1-BV605, Clone PK136, 108753, Biolegend
CD11b-BV711, Clone M1/70, 101242, BioLegend
CD11b-APC-Cy7, Clone ICRF44, 560914, BD Biosciences
CD4-FITC, Clone RM4-5, 100510, BioLegend
CD4-FITC, Clone GK1.5, 100406, Biolegend
CD8-BV421, Clone 53-6.7, 100738, BioLegend
F4/80-APC, Clone BM8, 17-4801-82, Invitrogen
Ly6G-PE, Clone 1A8, 127608, BioLegend
Ly-6G- PE-Cy7, Clone 1A8, 560601, BD Biosciences
Ly-6C- PerCP/Cy5.5, Clone HK1.4, 128012, Biolegend
IA/IE-BV786, Clone M5/114.15.2, 742894, BD Biosciences

Antibodies for T-cell depletion: (Antibody, Clone number, Cat no, Supplier)
InVivoMAb Rat IgG2b Isotype control, Clone LTF2, BE0090, BioXcell
InVivoMAb Anti- mouse CD4, Clone GK1.5, BE0003-1, BioXcell
InVivoMAb Anti-mouse CD8 alpha, Clone 2.43, BE0061, BioXcell

Validation

All antibodies used in this study are commercially available and were validated by the manufacturer. Links to the product page for all the antibodies are provided below. Antibodies used for Western blotting were validated in our study by presence of band(s) corresponding to predicted molecular weight compared to protein ladder in positive and negative control samples. Flow cytometry antibodies were validated by the manufacturer and were validated in our study using known positive vs negative populations (e.g. B cells do not express CD8).

Antibodies for Western blotting:
- Actin, 4967, Cell Signaling (https://www.cellsignal.com/products/primary-antibodies/b-actin-antibody/4967)
- ATRX (Human specific), Clone D1N2E, 14820, Cell Signaling (https://www.cellsignal.com/products/primary-antibodies/atrx-d1n2e-rabbit-mab/14820)
- ATRX, NBP1-32851, Novus Biologicals (https://www.novusbio.com/products/atrx-antibody_nbp1-32851)
- ATRX, Clone E5X7O, 10321, Cell Signaling (https://www.cellsignal.com/products/primary-antibodies/atrx-e5x7o-rabbit-mab/10321)
- IDH1 R132H, Clone H09, DIA-H09, Dianova (https://www.dianova.com/en/shop/dia-h09-anti-idh1-r132h-hu-from-mouse-h09-unconj/)
- IDH1, Clone D2H1, 8137, Cell signaling (https://www.cellsignal.com/products/primary-antibodies/idh1-d2h1-rabbit-mab/8137)
- Phospho IRF3 (Ser396), Clone 4D4G, 4947, Cell signaling (https://www.cellsignal.com/products/primary-antibodies/phospho-irf-3-ser396-4d4g-rabbit-mab/4947)
- Phospho IRF3 (Ser396), Clone E.875.8, MA5-14947, Thermo scientific (https://www.thermofisher.com/antibody/product/Phospho-IRF3-Ser396-Antibody-clone-E-875-8-Monoclonal/MA5-14947)
- Total IRF3 (Human specific), Clone D9J5Q, 10949, Cell signaling (https://www.cellsignal.com/products/primary-antibodies/irf-3-d9j5q-mouse-mab/10949)
- Total IRF3, Clone 12A4A35, 655702, Biolegend (https://www.biolegend.com/en-ie/search-results/purified-anti-irf3-antibody-8629)
- Phospho STAT1 (Tyr701), Clone 58D6, 9167, Cell signaling (https://www.cellsignal.com/products/primary-antibodies/phospho-stat1-tyr701-58d6-rabbit-mab/9167)
- STAT1, 9172, Cell signaling (https://www.cellsignal.com/products/primary-antibodies/stat1-antibody/9172)
- STAT1, Clone D1K9Y, 14994, Cell signaling (https://www.cellsignal.com/products/primary-antibodies/stat1-d1k9y-rabbit-mab/14994)
- TBK1, Clone D1B4, 3504, Cell signaling (https://www.cellsignal.com/products/primary-antibodies/tbk1-nak-d1b4-rabbit-mab/3504)
- ISG15, 2743, Cell signaling (https://www.cellsignal.com/products/primary-antibodies/isg15-antibody/2743)
- RIG-I, Clone D14G6, 3743, Cell signaling (https://www.cellsignal.com/products/primary-antibodies/rig-i-d14g6-rabbit-mab/3743)
- MDA5, Clone D74E4, 5321, Cell signaling (https://www.cellsignal.com/products/primary-antibodies/mda-5-d74e4-rabbit-mab/5321)
- Vinculin, Clone hVIN-1, V9264, Sigma (https://www.sigmaaldrich.com/US/en/product/sigma/v9264)
- HRP conjugated beta-actin, Clone 13E5, 5125, Cell signaling (https://www.cellsignal.com/products/antibody-conjugates/b-actin-13e5-rabbit-mab-hrp-conjugate/5125)
- HRP conjugated beta- tubulin, Clone 9F3, 5346, Cell signaling (https://www.cellsignal.com/products/antibody-conjugates/b-tubulin-9f3-rabbit-mab-hrp-conjugate/5346)
- Anti-rabbit IgG, HRP conjugated antibody, 7074, Cell signaling (https://www.cellsignal.com/products/secondary-antibodies/anti-rabbit-igg-hrp-linked-antibody/7074)
- Anti-mouse IgG, HRP conjugated antibody, 7076, Cell signaling (https://www.cellsignal.com/products/secondary-antibodies/anti-

mouse-igg-hrp-linked-antibody/7076)

Antibodies for IHC:
- ATRX, Clone E5X7O, 10321, Cell signaling (https://www.cellsignal.com/products/primary-antibodies/atrx-e5x7o-rabbit-mab/10321)
- CD3 epsilon, Clone E4T1B, 78588, Cell Signaling (https://www.cellsignal.com/products/primary-antibodies/cd3e-e4t1b-xp-rabbit-mab/78588)
- CD45, ab10558, Abcam (https://www.abcam.com/products/primary-antibodies/cd45-antibody-ab10558.html)
- F4/80, Clone BM8, 14-4801-82, Thermo scientific (https://www.thermofisher.com/antibody/product/F4-80-Antibody-clone-BM8-Monoclonal/14-4801-82)
- IDH1 R132H, Clone MRQ-67, 456R-34, Cell Marque (Sigma) (https://www.cellmarque.com/antibodies/CM/3658/IDH1-R132H_MRQ-67)

Antibodies for flow cytometry:
- CD45.2-BUV395, Clone 104, 564616, BD Biosciences (https://www.bdbiosciences.com/en-us/products/reagents/flow-cytometry-reagents/research-reagents/single-color-antibodies-ruo/buv395-mouse-anti-mouse-cd45-2.564616)
- CD45-BUV395, Clone 30-F11, 564279, BD Biosciences (https://www.bdbiosciences.com/en-us/products/reagents/flow-cytometry-reagents/research-reagents/single-color-antibodies-ruo/buv395-rat-anti-mouse-cd45.564279)
- CD3-FITC, Clone 17A2, 100204, BioLegend (https://www.biolegend.com/en-ie/products/fitc-anti-mouse-cd3-antibody-45)
- CD3-PE, Clone 17A2, 100205, BioLegend (https://www.biolegend.com/en-ie/products/pe-anti-mouse-cd3-antibody-47)
- CD19-FITC, Clone 1D3/CD19, 152404, BioLegend (https://www.biolegend.com/en-ie/products/fitc-anti-mouse-cd19-antibody-13615)
- NK1.1-BV421, Clone PK136, 108732, BioLegend (https://www.biolegend.com/en-ie/products/brilliant-violet-421-anti-mouse-nk-1-1-antibody-7150)
- NK1.1-BV605, Clone PK136, 108753, Biolegend (https://www.biolegend.com/en-ie/products/brilliant-violet-605-anti-mouse-nk-1-1-antibody-8665)
- CD11b-BV711, Clone M1/70, 101242, BioLegend (https://www.biolegend.com/en-ie/products/brilliant-violet-711-anti-mouse-human-cd11b-antibody-7927)
- CD11b-APC-Cy7, Clone ICRF44, 560914, BD Biosciences (https://www.bdbiosciences.com/en-us/products/reagents/flow-cytometry-reagents/research-reagents/single-color-antibodies-ruo/apc-cy-7-mouse-anti-human-cd11b.560914)
- CD4-FITC, Clone RM4-5, 100510, BioLegend (https://www.biolegend.com/en-ie/products/fitc-anti-mouse-cd4-antibody-480))
- CD4-FITC, Clone GK1.5, 100406, Biolegend (https://www.biolegend.com/en-ie/products/fitc-anti-mouse-cd4-antibody-248)
- CD8-BV421, Clone 53-6.7, 100738, BioLegend (https://www.biolegend.com/en-ie/products/brilliant-violet-421-anti-mouse-cd8a-antibody-7138)
- F4/80-APC, Clone BM8, 17-4801-82, Thermo scientific (https://www.thermofisher.com/antibody/product/F4-80-Antibody-clone-BM8-Monoclonal/17-4801-82)
- Ly6G-PE, Clone 1A8, 127608, BioLegend (https://www.biolegend.com/en-ie/products/pe-anti-mouse-ly-6g-antibody-4777)
- Ly-6G- PE-Cy7, Clone 1A8, 560601, BD Biosciences (https://www.bdbiosciences.com/en-us/products/reagents/flow-cytometry-reagents/research-reagents/single-color-antibodies-ruo/pe-cy-7-rat-anti-mouse-ly-6g.560601)
- Ly-6C- PerCP/Cy5.5, Clone HK1.4, 128012, Biolegend (https://www.biolegend.com/en-ie/products/percp-cyanine5-5-anti-mouse-ly-6c-antibody-5967)
- IA/IE-BV786, Clone M5/114.15.2, 742894, BD Biosciences (https://www.bdbiosciences.com/en-us/products/reagents/flow-cytometry-reagents/research-reagents/single-color-antibodies-ruo/bv786-rat-anti-mouse-i-a-i-e.742894)

Antibodies for T-cell depletion:
- InVivoMAb Rat IgG2b Isotype control, Clone LTF2, BE0090, BioXcell (https://bioxcell.com/invivomab-rat-igg2b-isotype-control-anti-keyhole-limpet-hemocyanin-be0090)
- InVivoMAb Anti- mouse CD4, Clone GK1.5, BE0003-1, BioXcell (https://bioxcell.com/invivomab-anti-mouse-cd4-be0003-1)
- InVivoMAb Anti-mouse CD8 alpha, Clone 2.43, BE0061, BioXcell (https://bioxcell.com/invivomab-anti-mouse-cd8-alpha-be0061)

Eukaryotic cell lines

Policy information about cell lines and Sex and Gender in Research

Cell line source(s)	CT2A (Peter Fecci, Duke University Medical Center) - female origin<br>CT2A CRISPR control, Atrx KO-A, Atrx KO-B (generated in this study from CT2A parental line)<br>CT2A CRISPR control/ MSCV empty vector control, CRISPR control/ MSCV Idh1-R132H, Atrx KO-A/ MSCV empty vector control, Atrx KO-A/ MSCV Idh1-R132H, Atrx KO-B/ MSCV empty vector control, Atrx KO-B/ MSCV Idh1-R132H (generated in this study)<br>M059J (ATCC)<br>293FT (Thermo Scientific)<br>UMNSAH/DF1 - (ATCC)<br>RCAS-PDGFa-shp53 expressing DF1 (generated in this study from parental DF1)<br>RCAS-Cre expressing DF1 (generated in this study from parental DF1)<br>RCAS/Ntv-a Atrx+/+ or Atrx-/- lines - generated in this study, both from a male mouse
Authentication	CT2A was confirmed to be of C57BL/6 origin by whole exome sequencing. 293FT was not independently authenticated by our group. M059J was authenticated using Cytochrome C subunit I (COI) PCR assay and STR profiling by ATCC.<br>Absence of full length ATRX protein was confirmed by immunoblotting in ATRX KO lines generated in this study. Expression of IDH1-R132H in CT2A mutant IDH1 lines was confirmed by immunoblotting and immunofluorescence.<br>Untransduced UMNSAH/DF1 cells were not independently authenticated in this study.

	RCAS-PDGFa-shp53 expressing DF1 cells were validated by western blotting for PDGFa.<br>RCAS-Cre expressing DF1 cells were validated by western blotting for Cre recombinase.<br>RCAS Ntv-a Atrx KO authentication was confirmed by absence of ATRX on western blotting.
Mycoplasma contamination	All cell lines tested negative for mycoplasma during routine testing using either the MycoAlert® PLUS kit (Lonza) or the Mycoplasma PCR Detection Kit (ABM, cat G238).
Commonly misidentified lines<br>(See ICLAC register)	No commonly misindentified lines were used in this study.

Animals and other research organisms

Policy information about studies involving animals; ARRIVE guidelines recommended for reporting animal research, and Sex and Gender in Research

Laboratory animals	Female nude (JAX# 002019) or C57BL/6 mice (JAX #000664) aged 8 to 10 weeks were purchased from Jackson Laboratories (Bar Harbor, ME). Animals were housed in filter top cages in Thoren units within the Duke University Cancer Cell Isolation Facility (CCIF) with 12 hours light-dark cycles at temperature of 21 (+/- 3) degree C and a relative humidity of 30-70% . Food and water were provided ad libitum.<br>J12 (nestin-Tva bearing, JAX#003529) mice were generously provided by Dr. Eric Holland and ATRX fl/fl (ATRX tm1Rjg, MGI#3528480) mice were generously provided by Dr. David Picketts. Initial generation of RCAS tumors was done via injection of RCAS bearing cells into 1-5 day old mice. Xenograft experiments were conducted in mice at 8 to 10 weeks of age. Animals were housed in filter top cages in Tecniplast units within the MD Anderson North Campus Animal Facility, with a 12 hour light cycle from 6:00am- 6:00pm, at temperature of 22.2 degree C (+/- 2) and a relative humidity of 40% - 55% (Set point 45%) . Food and water were provided ad libitum.
Wild animals	No wild animals were used in this study
Reporting on sex	CT2A glioma line is of female origin and, therefore, only female mice were used for implants with CT2A lines.<br>Tumors were generated in both male and female RCAS-nTva mice, as approximately a 50/50 ratio. Cell lines reported were both of male origin, and xenografts were only performed in male mice for this reason.
Field-collected samples	No field collected samples were used in this study.
Ethics oversight	All animal study protocols were approved by and performed in accordance with Duke and MD Anderson Cancer Center Institutional Animal Care and Use Committee (IACUC) guidelines.

Note that full information on the approval of the study protocol must also be provided in the manuscript.

Plants

Seed stocks	N/A
Novel plant genotypes	N/A
Authentication	N/A

Flow Cytometry

Plots

Confirm that:

- [x] The axis labels state the marker and fluorochrome used (e.g. CD4-FITC).
- [x] The axis scales are clearly visible. Include numbers along axes only for bottom left plot of group (a 'group' is an analysis of identical markers).
- [x] All plots are contour plots with outliers or pseudocolor plots.
- [x] A numerical value for number of cells or percentage (with statistics) is provided.

Methodology

Sample preparation	As described in materials & methods, CT2A and RCAS mouse tumor-bearing hemispheres were minced and dissociated using Liberase and DNaseI to generate single cell suspensions. Any remaining tissue pieces were dissociated through a 70 micron

	cell strainer, washed in HBSS and subjected to myelin removal after overlaying on 20% Percoll in HBSS. Single cell suspensions were washed in PBS, stained with Zombie-Aqua, washed in FACs buffer [2% FBS in PBS], reconstituted in FACs buffer containing 1:50 Tru-stain FC block and stained with antibody mastermixes.<br><br>Conditioned media from untreated/ treated cell lines were used to measure cytokine levels using a LEGENDplex assay kit (BioLegend), as described in materials and methods.
Instrument	BD Fortessa X-20 flow cytometer (Duke Cancer Institute Flow Cytometry Core Facility; MD Anderson South Campus Flow Cytometry Core)
Software	BD FACS DIVA was used for data acquisition. Flow-Jo v10.8.1 (BD Biosciences) was used for data anlaysis.
Cell population abundance	Downstream T cell and macrophage abundance was determined after collection of a minimum of 20,000 live singlets for each sample.
Gating strategy	As shown in gating strategies provided in the supplementary information, T cells were defined as CD45+ CD3+ as a proportion of live singlets; with downstream gating for CD4+ vs CD8+ T cells. Macrophages were defined as CD45hi, CD11bhi after gating for live singlets. Live gates were determined using Zombie Aqua staining as shown in gating strategy.

[x] Tick this box to confirm that a figure exemplifying the gating strategy is provided in the Supplementary Information.
