# Supplementary material for: Interplay between ATRX and IDH1 mutations governs innate immune responses in diffuse gliomas
Source: Nat Commun. 2024 Jan 25;15:730. doi: 10.1038/s41467-024-44932-w (PMC10810843; doi:10.1038/s41467-024-44932-w)
Supplement: Supplementary file 3 — Description of Additional Supplementary Files [file 41467_2024_44932_MOESM3_ESM.pdf]

Title: Supplementary data 1 - TCGA frequency table (Excel file):
Description: Frequency table of results from single-sample gene set enrichment analysis (ssGSEA) of TCGA bulk RNAseq datasets from IDH mutant low-grade gliomas - contains positive enrichment frequencies for the various immune-related GO pathways analyzed in these datasets.

Title: Supplementary data 2 –scRNAseq GSEA (Excel file):
Description: Contains results from GSEA performed on scRNAseq data from GEO datasets GSE89567 (IDH-mutant astrocytoma) and GSE70630 (IDH-mutant oligodendroglioma).

Title: Supplementary data 3 –RCAS tumors_RNAseq GSEA (Excel file):
Description: Contains results from GSEA performed on RNAseq data from RCAS/Ntv-a tumors.
